# Sex-dimorphic reprogramming of fetal mouse brain development by maternal estradiol excess

**DOI:** 10.1186/s13293-025-00792-7

**Published:** 2025-12-02

**Authors:** Huihui Wang, Zhe Wei, Yu Zhang, Xiaojun Chen, Li Jin, Chengliang Zhou

**Affiliations:** 1 Department of Reproductive Medicine, Shanghai Key Laboratory for Assisted Reproduction and Reproductive Genetics, Ren Ji Hospital, Shanghai Jiao Tong University School of Medicine, Shanghai, China; 2 Obstetrics and Gynecology Hospital, Institute of Reproduction and Development, Fudan University, Shanghai, China; 3 School of Medicine, International Peace Maternity and Child Health Hospital, Shanghai Jiao Tong University, Shanghai, China; 4 Key Laboratory of Embryo Original Diseases, Shanghai, China; 5 Research Units of Embryo Original Diseases, Chinese Academy of Medical Sciences, Shanghai, China

**Keywords:** Sex difference, Development programming, Maternal estradiol, Brain development, Spatial transcriptomics, Single-cell RNA sequencing

## Abstract

**Background:**

Gestational environmental perturbations can induce sex-specific developmental programming, increasing offspring susceptibility to chronic diseases. While prenatal high estradiol (HE) exposure has been associated with male-biased neurodevelopmental disorders, the underlying mechanisms remain poorly understood.

**Methods:**

Using spatial transcriptomics in a murine HE exposure model, we systematically characterized sex-divergent molecular and cellular responses in fetal brains. Through cell type identification, spatial mapping, ligand-receptor interaction analysis, and transcription factor activity assessment, we examined gene expression profile, intra-regional signaling pathway, and regulon activity variations. Additionally, we performed immunofluorescence to characterize neural progenitor cell dynamics.

**Results:**

Our analysis revealed that maternal HE exposure differentially altered gene expression patterns between male and female fetal brain regions, with more pronounced effects on male-biased genes. Notably, HE-induced downregulation of male-biased genes was proportional to their baseline male-bias degree. We uncovered region-specific cellular responses to HE exposure and demonstrated sex-opposed alterations in intra-regional signaling pathway. Furthermore, we identified cell type- and brain region-restricted sex differences in regulon activity variations. Histological validation confirmed that maternal HE exposure specifically disrupts the proliferation-differentiation balance of neural progenitor cells in the male cerebral cortex.

**Conclusions:**

These findings provide mechanistic insights into sex-dimorphic developmental reprogramming of fetal brain by maternal estradiol excess. They establish a framework for developing targeted interventions against gestational endocrine disruption-induced neurodevelopmental disorders.

**Supplementary Information:**

The online version contains supplementary material available at 10.1186/s13293-025-00792-7.

## Plain language summary

During pregnancy, certain environmental factors can affect how a baby’s brain develops, sometimes leading to long-term health problems. High levels of maternal estradiol (HE) have been linked to brain development issues in offspring, especially in males, but scientists don’t fully understand why. This study investigated how HE exposure affects male and female fetal brains differently in mice. It found that HE changed different gene expression in two sexes, with more changes in genes that are normally highly expressed in males. Interestingly, the more a gene was typically male-biased, the more HE reduced its expression. It also discovered that different brain regions and cell types reacted to HE in unique ways, with males and females showing opposite changes in brain cell communication and specific alterations in gene regulation activity. Most importantly, HE disrupted the growth and maturation of brain-building cells (neural progenitors) in a certain region of male brain, which might explain why males are more vulnerable. These findings help explain how excess estradiol during pregnancy may harm brain development differently in male and female offsprings, they could lead to better ways to prevent or treat developmental disorders caused by maternal hormone imbalances.

**Supplementary Information:**

The online version contains supplementary material available at 10.1186/s13293-025-00792-7.

## Highlights

- High maternal estradiol (HE) induces distinct alteration of transcriptional profile and sex-biased gene expression in male and female fetal brains.
- HE dysregulates ligand-receptor interactions and transcription factor activities in specific brain regions and cell types in a sex-dimorphic manner.
- HE disrupts proliferation-differentiation balance of neural progenitors in the male cerebral cortex.
- Findings reveal how maternal hormone imbalances sex-specifically reprogram fetal brain development, paving the way for targeted prevention strategies.

**Supplementary Information:**

The online version contains supplementary material available at 10.1186/s13293-025-00792-7.

## Background

The Developmental Origins of Health and Disease (DOHaD) theory, which establishes that prenatal conditions profoundly influence lifelong health trajectories, has emerged as a critical framework for understanding the etiology of chronic diseases [1]. A critical aspect of this paradigm is the recognition of sex-specific developmental programming, whereby environmental perturbations differentially affect male and female offspring. Epidemiological evidence demonstrates this sexual dimorphism across multiple systems: maternal stress preferentially increases female offspring’s risk for neuropsychiatric disorders [2], whereas adverse intrauterine environments predominantly predispose males to cardiovascular dysfunction [3]. Environmental toxicants similarly exhibit sex-biased effects on immune system development [4]. These divergent outcomes likely arise from dynamic interactions between sex-specific placental responses [5], fetal hormonal milieu [6], and postnatal environment factors [7]. Elucidating these mechanisms is paramount for developing targeted interventions against the growing burden of sex-dimorphic chronic diseases.

Assisted reproductive technologies (ART) have introduced novel considerations to the DOHaD framework. As a cornerstone treatment for infertility, ART has contributed to the expansion of the global population by 0.1% [8], necessitating rigorous evaluation of its developmental impacts. A key alteration in ART pregnancies is the supraphysiological estradiol levels resulting from ovarian stimulation during fresh embryo transfer, which persist through early gestation [9]. Although maternal estradiol is crucial for normal development, its dysregulation can have profound consequences [10]. Our previous research revealed that elevated maternal estradiol triggers sex-specific metabolic reprogramming, inducing male-specific insulin resistance through hypothalamic gene expression changes and impaired neurogenesis [11, 12]. This sex-specific vulnerability extends to neurodevelopmental outcomes, as evidenced by the association between elevated maternal estradiol and increased autism spectrum disorder (ASD) risk in male offspring [13, 14]. The biological basis for these disparities may lie in the fundamental role of estradiol in sexual differentiation of the brain, where it is essential for female neuroendocrine development [15] and male brain masculinization [16]. These findings suggest that the two sexes exhibit unequal vulnerabilities to the disruption of maternal estradiol in terms of neurodevelopment. Despite these insights, the genomic and cellular mechanisms mediating sex-specific responses to maternal estradiol remain elusive.

The sexually dimorphic organization of the brain, including differences in size [17], functional connections [18], and gene regulatory networks [19], likely drives their distinct responses to maternal estradiol. These differences originate from genomic biases. While the embryonic mouse brain transcriptome has been characterized [20], a comprehensive spatial and cellular resolution of sex-specific features is lacking. To address this knowledge gap, we employed 10× Genomics Visium spatial transcriptomics to systematically map sex-dimorphic responses to prenatal high estradiol (HE) exposure across major brain regions. By integrating spatial transcriptomic profiles with single-cell RNA sequencing (scRNA-seq) data, we delineated cellular interactions and regulatory networks altered by HE exposure. Our findings not only establish the sex-dimorphic impact of prenatal HE on brain development but also provide a mechanistic framework for understanding fetal programming of sex-biased neurodevelopmental disorders, offering potential avenues for targeted interventions.

## Methods

### Animal model and ethical approval

A mouse model of prenatal HE exposure was established following our previously published protocol [11]. Briefly, 8-week-old pregnant C57BL/6 mice were purchased from Slaccas Laboratory Animal (Shanghai, China) and administered 100 µg/kg/d estradiol valerate (MCE, catalog no. HY-B0672, New Jersey, USA) (HE group) dissolved in corn oil or an equal volume of corn oil only (vehicle control, VC group) via gavage from E5.5 to E11.5. At E18.5, the fetuses were harvested and decapitated. Fetal sex was determined by microscopic examination of gonadal morphology (testes in males, ovaries in females). Brains were rapidly dissected and either embedded in optimal cutting temperature (OCT) compound for spatial transcriptomics or fixed in 4% paraformaldehyde for paraffin embedding and immunofluorescence analysis. To minimize the litter effects, a total of 10 pregnant C57BL/6 mice were used, with 5 assigned to the VC group and 5 to the HE group. From each dam, one male and one female fetus were randomly selected for spatial transcriptomics and histological staining, resulting in a total of 10 fetuses per group (5 males and 5 females). One fetus per sex per group were randomly selected for spatial transcriptomics, three out of the rest four fetuses per sex per group were randomly selected for histological staining. The randomization was performed using a lottery method. No animals were excluded in the analysis. All procedures were approved by the Institutional Animal Care and Use Committee of Shanghai Model Organisms Center (Shanghai, China) (permit no. 2022-0051-1) and conducted in accordance with relevant ethical guidelines.

## Tissue processing and spatial transcriptomics

OCT-embedded brains were cryosectioned at −20 °C into 10 μm coronal sections using a Leica 3050 S Cryostat. Thalamic coronal sections within the scope of positions 258 to 268 of the E18.5 Allen Brain Atlas were mounted on the capture area of the 10× Genomics Visium (Pleasanton, USA) slide. Following methanol fixation and hematoxylin-eosin (H&E) staining (10× Genomics protocol CG000160), tissue permeabilization was optimized to 18 min for maximal mRNA capture. The RNA sample quality was assessed using Agilent Bioanalyzer (Santa Clara, USA) prior to sequencing. Spatial gene expression libraries were prepared through reverse transcription and second-strand cDNA synthesis, followed by paired-end sequencing on an Illumina NovaSeq 6000 (San Diego, USA) platform. Data processing was performed using Space Ranger software (v1.0.0) to generate feature-spot matrices and perform clustering analysis. The reads per spot ranged from 143,676 to 242,176. The unique molecular identifier (UMI) count matrix was normalized using the SCTransform algorithm in Seurat (v3.1.1). Dimensionality reduction was achieved through principal component analysis (PCA), with results visualized using Uniform Manifold Approximation and Projection (UMAP) [21]. The Seurat’s FindAllMarker module was utilized to analyze the standardized expression data and detect the cluster-specific marker genes (padj < 0.05, Benjamini-Hochberg FDR [22], min.pct = 0.5).

## Brain region identification

The E18.5 Allen Brain Atlas (accessed : August 2024) [23] integrated with The Brain Explorer 2 software (accessed : August 2024) [24] was used to ascertain the corresponding brain region of each cluster in spatial transcriptomics. Regional assignments are illustrated in Figure S1.

## HE-induced DEG identification and functional enrichment analysis

Differentially expressed genes (DEGs) between HE and VC groups in each cluster were identified using Seurat’s FindMarker module through Wilcoxon rank-sum tests [25], with significance thresholds set at *p* < 0.05 and |log_2_foldchange| >0.26. Clustering and Gene Ontology (GO) enrichment analysis was performed and visualized using the ClusterGVis package (v0.1.3) (clustered using the k-means algorithm).

## Sex-biased and HE-affected sex-biased DEG analysis

Sex-biased DEGs were identified by comparing gene expression between male and female control within each brain region (*p* < 0.05, |log_2_foldchange| >0.5). HE-affected sex-biased DEGs were defined as the intersection between HE-induced DEGs and sex-biased DEGs. The average expression values of the DEGs were calculated using the AverageExpression() function in Seurat, stratified by clusters and groups. A heatmap was generated using the pheatmap package (v1.0.12), with values standardized as z-scores through the scale parameter for normalization. Cell type-specific networks were constructed in Cytoscape (v3.7.2).

### Immunofluorescence and TUNEL assay

Paraffin-embedded brain Sect. (4 μm) were deparaffinized, rehydrated, and subjected to antigen retrieval in EDTA buffer (pH 9.0) using a microwave oven. Sections were blocked (1% goat serum, 1% BSA in PBS) and incubated with primary antibodies at 4 °C overnight, followed by horseradish peroxidase (HRP)-conjugated secondary antibodies and tyramide signal amplification. The slides were heat-treated after each tyramide signal amplification and the nuclei were counterstained with 4’,6-diamidino-2-phenylindole after all antigens had been labeled. Primary antibodies included anti-TTR (1:200, Proteintech, catalog no. 11891-1-AP, Chicago, USA), anti-MARCKS (1:100, Proteintech, catalog no. 10004-2-Ig, Chicago, USA), anti-TNC (1:100, Abcam, catalog no. ab108930,Cambridge, UK), anti-PAX6 (1:100, BioLegend, catalog no. 901302, San Diego, USA), anti-PCNA (1:200, Proteintech, catalog no. 10205-2-AP, Chicago, USA), anti-TBR2 (1:200, eBioscience, catalog no. 14–4875-80, San Diego, USA), and anti-DCX (1:200, Proteintech, catalog no. 13925-1-AP, Chicago, USA). Terminal deoxynucleotidyl transferase dUTP nick-end labeling (TUNEL) staining was performed using a commercial kit (Roche, catalog no. 11684817910, Basel, Switzerland) following manufacturer protocols. Cell number counting and staining area measurement were performed using Image J (v1.54 m, NIH).

## Cell type identification and RCTD analysis

ScRNA-seq data from publicly available sources (accessed: August 2024) [26] were used to identify nine cell types through unsupervised clustering (the original Louvain algorithm in the Seurat’s FindClusters module). The Seurat’s FindAllMarker module was utilized to analyze the standardized expression data and detect the cluster-specific marker genes (padj < 0.05, Benjamini-Hochberg FDR [22], min.pct = 0.5). The cell type of each cluster was annotated according to their expression of the known canonical marker genes. Cell type assignment in spatial transcriptomics spots was performed using the Robust Cell Type Decomposition (RCTD) method (v1.1.0) [27] with default parameters, except for minimum cell (1) and UMI (1) requirements per spot. Doublet detection was disabled (doublet_mode = FALSE) to infer spot-specific cell type composition.

## Ligand-receptor interaction analysis

Ligand-receptor interaction was analyzed using CellChat (v2.1.0) [28, 29]. Normalized expression matrices were processed using identifyOverExpressedGenes, identifyOverExpressedInteractions, and projectData functions. Potential ligand-receptor interactions were calculated using the computeCommunProb, filterCommunication (min.cells = 10) and computeCommunProbPathway functions, with networks aggregated via aggregateNet.

### Transcription factor activity analysis

Single-Cell Regulatory Network Inference and Clustering (SCENIC) (v1.1.2) [30] was used to assess transcription factor activity, utilizing the RcisTarget motifs database (v1.2.1) and GRNboost2 with default parameters. Co-expression-based target gene identification and motif analysis were performed using RcisTarget. Regulon activity score(RAS)were calculated using AUCell (v1.4.1). Regulon specificity was evaluated through regulon specificity scores (RSS) [31] and connection specificity indices (CSI) using scFunctions.

### Gene score measurement

Gene set scores in specific pathways and regulons were calculated using Seurat’s AddModuleScore function, which computes average expression levels of target genes relative to control feature sets. Control features were randomly selected from expression-based bins.

### Statistical analysis

The majority of data analysis was performed using R programming language (version 4.0.3). The key R packages and their versions used for specific analyses were specified in the relevant method subsections. Pearson correlation was used to assess relationships between sex bias and HE’s effect. Pearson’s chi-squared test compared proportions of spatial spots with positive RAS. Immunofluorescence signal comparisons used Student’s *t*-tests. Statistical significance was set at *p* < 0.05.

## Results

### Prenatal HE induces sex-divergent transcriptional changes in fetal brains

**Fig. 1:**
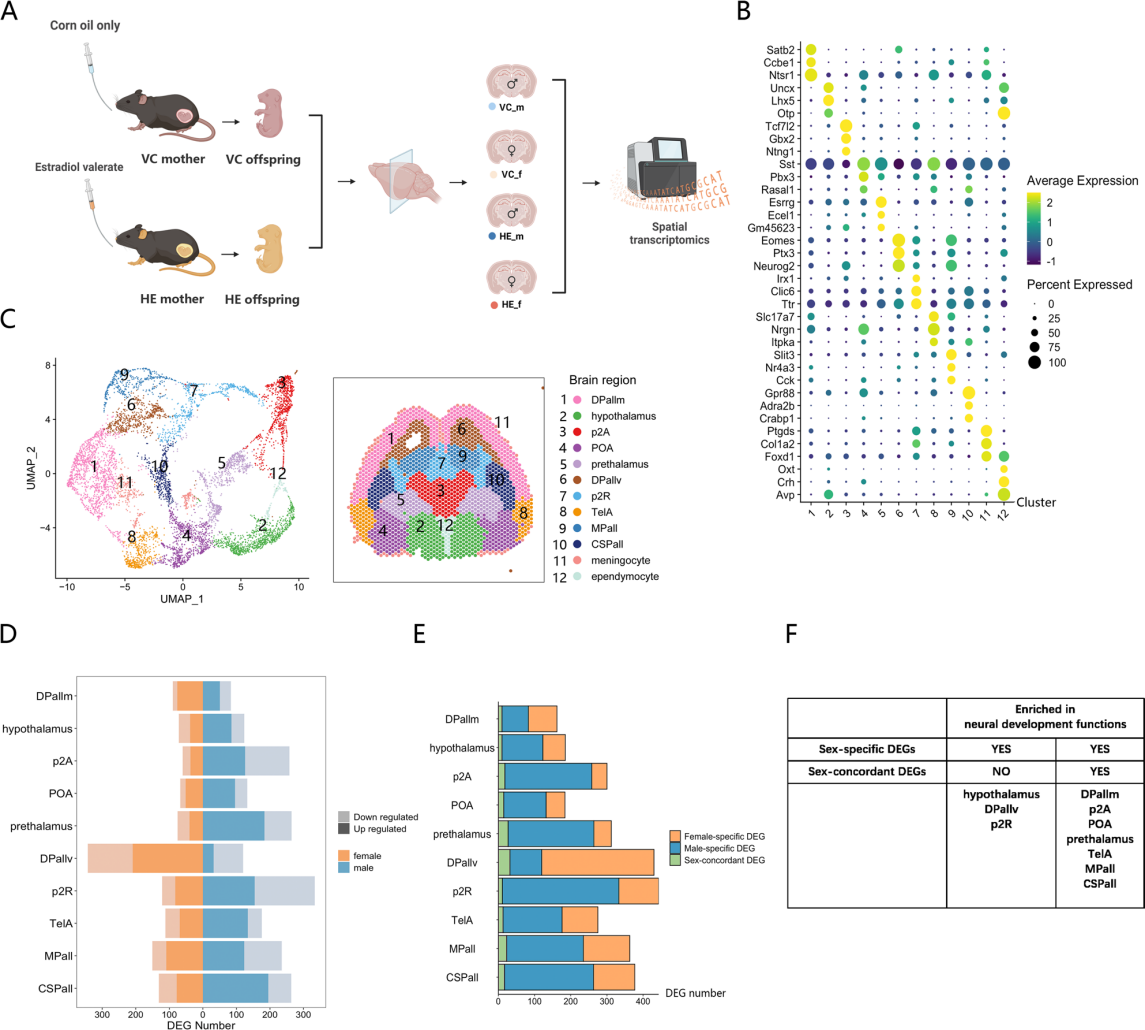
Spatial transcriptomic profiling of fetal brains in prenatal HE mouse model. (**A**) Experimental workflow for fetal brain section preparation and spatial transcriptomic analysis. (**B**) Dot plot of cluster-specific marker genes, with colors representing the average expression and diameters indicating the percentage of spots expressing the marker genes. (**C**) UMAP visualization of transcriptional profiles with spatial clustering annotations aligned to anatomical brain regions in fetal brain sections. (**D**) Number of HE-induced DEGs across brain regions. (E) Number of sex-specific versus sex-concordant DEGs across brain regions. (**F**) Table demonstrating the association between HE-induced DEG categories and neural development-related GO terms in each brain region

To examine the impact of prenatal HE exposure, we established HE-exposed and VC mouse models following published protocols [11]. Male (VC_m and HE_m) and female (VC_f and HE_f) fetal brains were dissected at embryonic day 18.5 (E18.5). Coronal brain sections were analyzed using spatial transcriptomics (Fig. 1A), yielding 1,859–1,979 spots per section, with an average of 2,252–2,430 genes per spot. Clustering with the original Louvain algorithm identified 12 clusters per slide, revealing specific marker genes for each cluster (Fig. 1B; Table S1). Using the E18.5 Allen Brain Atlas [23] and Brain Explorer 2 software [24], we annotated these clusters as: dorsal pallium mantle zone (DPallm), hypothalamus, p2 alar plate (p2A), preoptic alar plate (POA), prethalamus, ventricular zone of dorsal pallium (DPallv), p2 roof plate (p2R), telencephalic vesicle alar plate (TelA), medial pallium (MPall), central subpallium (CSPall), meningocytes, and ependymocytes (Fig. 1C; Figure S1). Meningocytes and ependymocytes were excluded from further analysis due to their single-cell-layer structure and compromised tissue integrity.

**Fig. 2:**
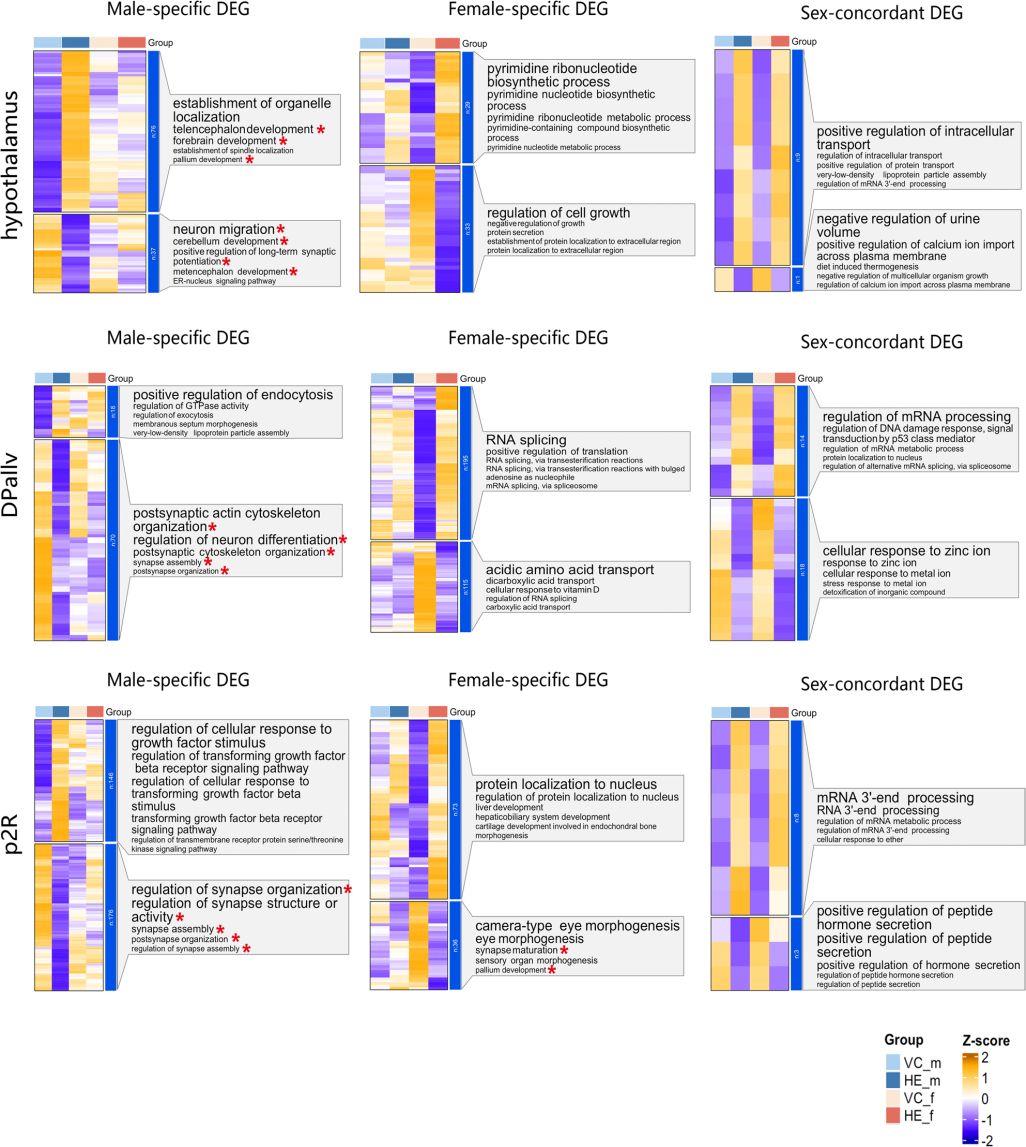
Functional enrichment of HE-induced DEGs. GO enrichment analysis of sex-specific and sex-concordant DEGs in hypothalamus, DPallv and p2R, asterisks indicate neural development-related functions

Comparative analysis revealed significant sex differences in the number of HE-induced DEGs across brain regions (Fig. 1D; Table S2). To characterize these sex-associated transcriptional changes in neural development, we classified them into two categories: sex-specific DEGs (changed in one sex only or changed in both sexes with opposite directions) and sex-concordant DEGs (changed in both sexes with the same direction) (Fig. 1E) for subsequent GO analysis, focusing particularly on neural development-related terms (Table S3). Notably, in hypothalamus, DPallv and p2R, neural development-related enrichment was exclusively observed in sex-specific DEGs but absent in sex-concordant DEGs (Figs. 1F and 2). In contrast, other brain regions exhibited neural development-related enrichment in both sex-specific and sex-concordant DEGs (Fig. 1F; Figure S2; Figure S3). These findings demonstrated brain region-specific sexual disparity in the potential neural developmental consequences of HE-induced transcriptional changes.

### Prenatal HE regulates sex-biased gene expression in fetal brains

**Fig. 3:**
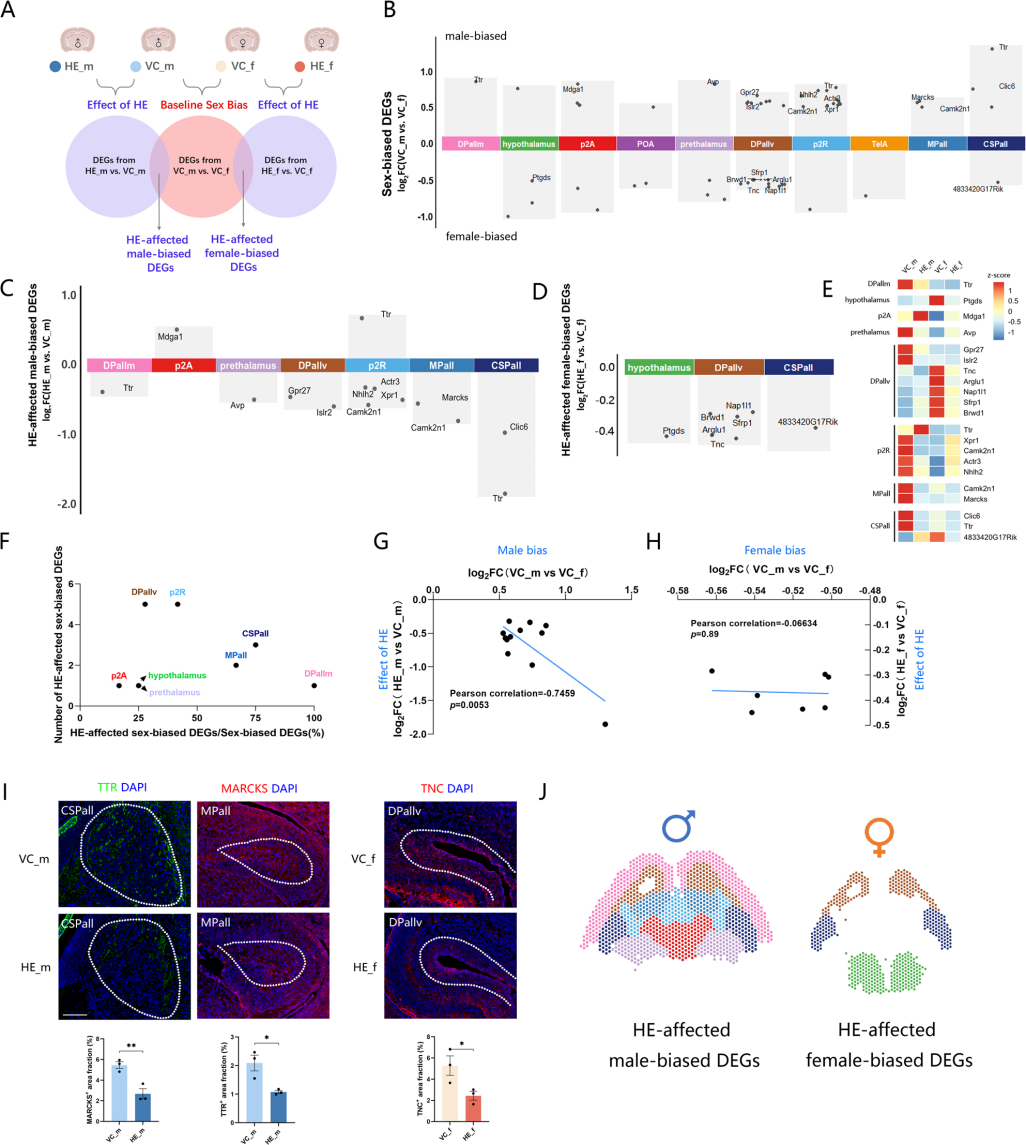
Sex-biased and HE-affected sex-biased DEG expression patterns. (**A**) Schematic workflow for identifying HE-affected sex-biased DEG, including HE-affected male- and female-biased DEGs. (**B**) Distribution of male- and female-biased DEGs across brain regions. (**C**, **D**) HE-affected male-biased (**C**) and female-biased (**D**) DEGs in each brain region. (**E**) Heatmap of HE-affected male- and female-biased DEGs expression, with colors of the blocks representing z-sore. (**F**) Quantification and proportion of HE-affected sex-biased DEGs relative to total sex-biased DEGs. (**G**, **H**) Correlation between log_2_foldchange of HE-induced and sex-biased DEGs in males (**G**) and females (**H**). (**I**) Top: Representative immunofluorescence images of TTR, MARCKS and TNC in corresponding brain regions (Scale bar: 200 μm). Bottom: Quantification of positive staining area fraction. Data are presented as mean ± SEM, significance was determined by Student’s t-test. *p < 0.05; **p < 0.01. (J) Brain region localization of HE-affected male-and female-biased DEGs

To investigate the relationship between intrinsic sex differences and sex-specific responses to HE exposure, we analyzed gene expression patterns by comparing baseline sex differences (VC_m vs. VC_f) and HE-induced changes (HE_m vs. VC_m, HE_f vs. VC_f). We then defined HE-affected male-biased DEGs (overlap between VC_m vs. VC_f and HE_m vs. VC_m) and HE-affected female-biased DEGs (overlap between VC_m vs. VC_f and HE_f vs. VC_f) (Fig. 3A). Male-biased and female-biased DEGs, which exhibited higher expression in male or female VC brains, were unevenly distributed across brain regions (Fig. 3B). 86% (12/14) of male-biased DEGs and 100% (7/7) of female-biased DEGs were downregulated by HE exposure (Fig. 3C, D), their exact log_2_foldchange values can be found in Table S2. The expression values of these HE-affected sex-biased DEGs were illustrated in a heatmap (Fig. 3E). The ratio of HE-affected sex-biased DEGs to total sex-biased DEGs was quantified (Fig. 3F). Notably, HE-induced downregulation of male-biased genes appeared to show a proportional relationship with their baseline male-bias degree, whereas female-biased DEGs exhibited no such correlation (Fig. 3G, H). To validate our transcriptomic findings at the protein level, we selected the most prominently dysregulated HE-affected sex-biased DEGs (TTR, MARCKS, and TNC) that were also expressed in distinct brain regions to assess the spatial extent of HE effects. Immunofluorescence validation confirmed the expression patterns of these three representative DEGs (Fig. 3I). Spatial distribution of all HE-affected sex-biased DEGs is presented in Fig. 3J.

**Fig. 4:**
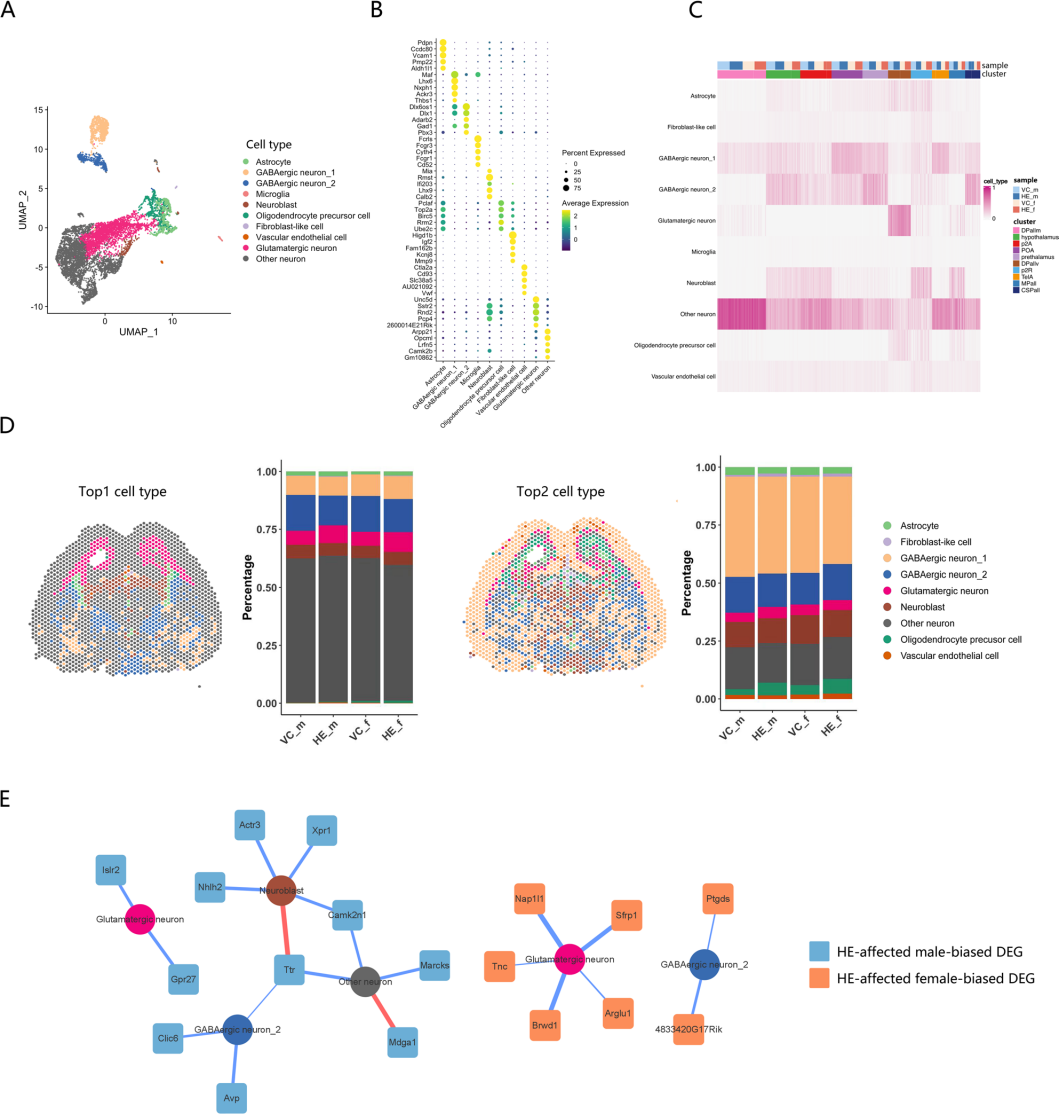
Single-cell characterization of fetal brain cell types and their association HE-affected sex-biased gene networks. (**A**) UMAP visualization of the scRNA-seq dataset from E18 mouse brains and identified cell types. (**B**) Dot plot of cell type-specific marker genes, with colors representing the average expression and diameters indicating the percentage of cells expressing the marker genes. (**C**) Heatmap of cell type composition across brain regions. (**D**) Spatial mapping of top1 and top2 predominant cell types in each spot, with quantitative composition analysis across experimental groups. (**E**) Networks of HE-affected male- and female-biased DEGs with their predominant cell type within each brain region, with nodes representing cell types and DEGs and edge widths weighted by log_2_foldchange values (from Fig. 3C and D; positive: red; negative: blue)

To delineate cell type-specific contributions to the sexually dimorphic responses, we integrated a scRNA-seq dataset of an E18 mouse brain with published marker genes [20, 32] to classify fetal brain cell populations, including astrocytes, GABAergic neurons (two subtypes), microglia, neuroblasts, oligodendrocyte precursor cells (OPCs), fibroblast-like cells, vascular endothelial cells, glutamatergic neurons, and other neurons (Fig. 4A, B; Figure S4; Table S4; Table S5). Spatial mapping of these cell types was performed using RCTD [27] (Table S6). The composition of cell types in each brain region was found to be uniformly distributed across all slices (Fig. 4C). Visualization of the two most probable cell types per spot demonstrated consistent composition and proportions among four groups (Fig. 4D).

Further analysis associated HE-affected sex-biased DEGs with the predominant cell types in each brain region (Fig. 4E). HE-affected male-biased DEGs were predominantly expressed in neuroblasts, glutamatergic neurons, GABAergic neurons_2, and other neurons, while HE-affected female-biased DEGs were mainly expressed in glutamatergic neurons and GABAergic neurons_2. These cell type-specific expression patterns highlight the intricate sexual dimorphism in cellular responses to prenatal HE exposure.

### Prenatal HE alters intra-regional signaling pathway in a sex-opposed manner

**Fig. 5:**
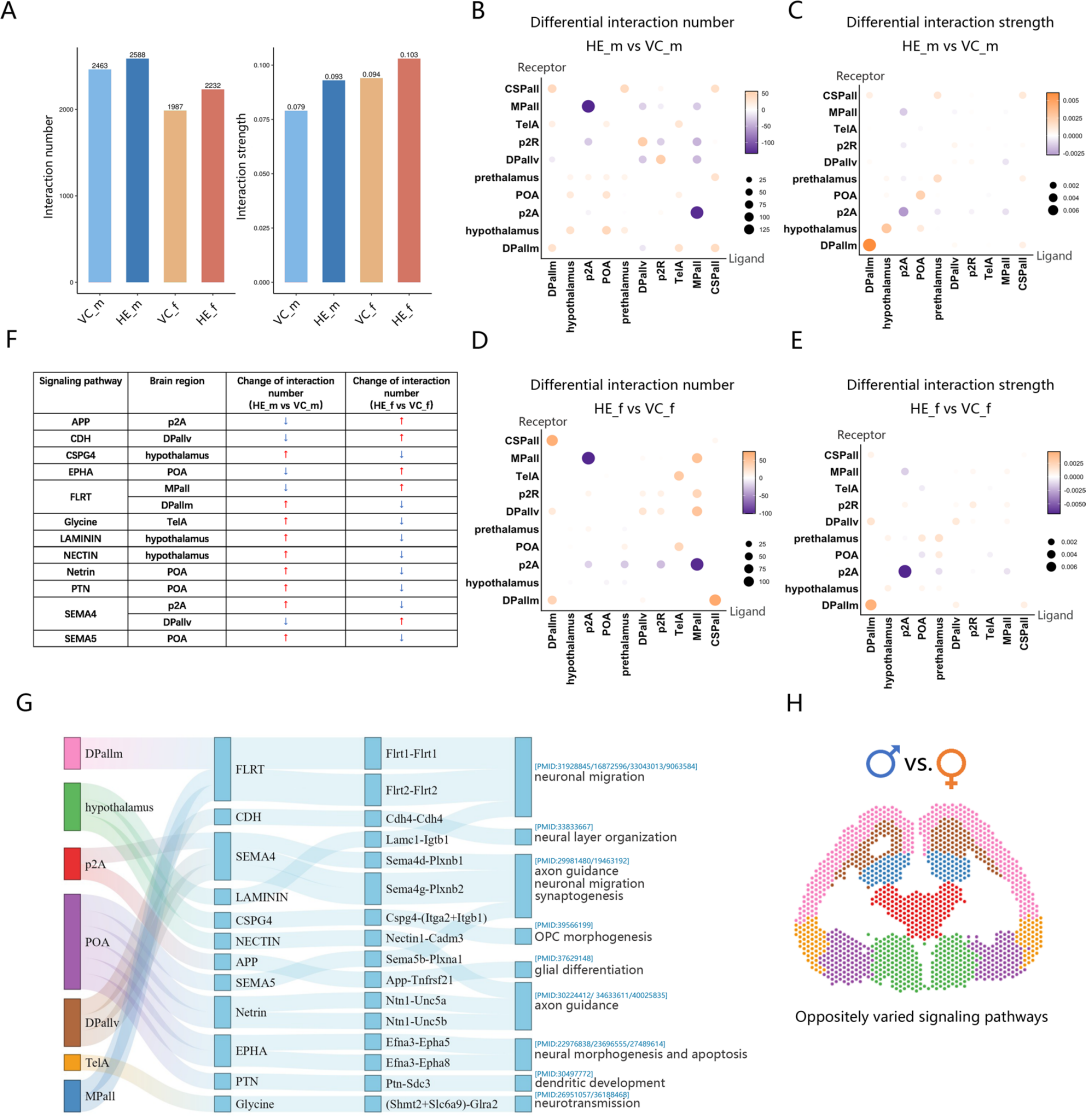
Sex-opposed signaling pathway changes identified by CellChat analysis. (**A**) Total ligand-receptor interaction number and strength across four experimental groups. (**B**, **C**) Differential ligand-receptor interaction number (**B**) and strength (**C**) in HE_m vs. VC_m brains (yellow: up-regulation; purple: down-regulation; spot size: magnitude of change). (**D**, **E**) Differential ligand-receptor interaction number (**D**) and strength (**E**) in HE_f vs. VC_f. brains (yellow: up-regulation; purple: down-regulation; spot size: magnitude of change). (**F**) Signaling pathways showing opposite changes in male and female HE vs. VC brains. (**G**) Integration of brain regions, oppositely varied signaling pathways, key ligand-receptor pairs and their established roles in neural development from literature evidence. (**H**) Brain region localization of oppositely varied pathways in two sexes

Given the crucial role of signaling pathways in brain region specialization, we investigated the sex-specific effects of prenatal HE exposure on intra-regional signaling networks. Using CellChat, we constructed ligand-receptor interaction networks (Table S7). Both male and female HE-exposed brains demonstrated enhanced interaction complexity, as evidenced by increased interaction numbers and strength (Fig. 5A). We mapped detailed inter- and intra-regional interaction networks (Fig. 5B-E) encompassing 52 signaling pathways (Figure S5).

Focusing on intra-regional alterations, we observed divergent changes in ligand-receptor interaction numbers. Strikingly, 12 pathways across seven brain regions showed opposing regulation trends between male and female HE-exposed brains compared to controls (Fig. 5F; Figure S6). We systematically visualized the network relationships among these brain regions, signaling pathways, key ligand-receptor pairs, and their neural developmental functions (Fig. 5G). Pathway gene scores were spatially mapped to corresponding regions (Figure S7). These oppositely varied signaling pathways are graphically localized (Fig. 5H).

### Prenatal HE differently alters Regulon activity in male and female fetal brains

**Fig. 6:**
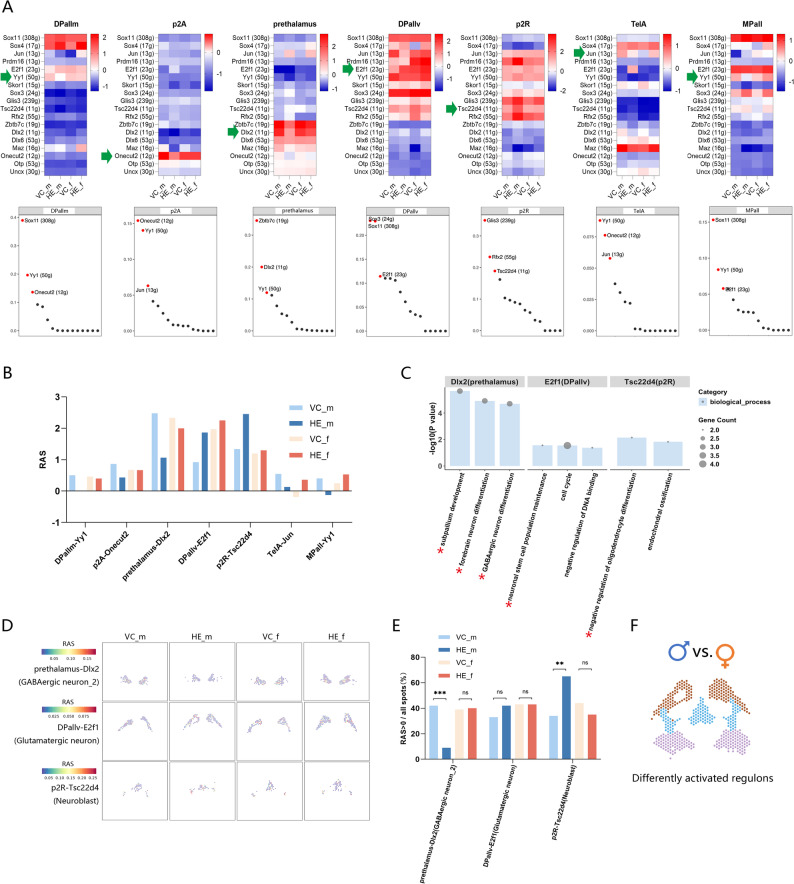
Sex-dimorphic regulon activity alterations revealed by SCENIC analysis. (**A**) Top: RAS heatmap of brain regions presenting sex-dimorphic RAS alterations, arrows indicate dimorphically activated regulons among the top three brain region-specific regulons. Bottom: Top three brain region-specific regulons ranked by RSS. (**B**) RAS of regulons showing sex-dimorphic alterations in HE vs. VC comparisons. (**C**) GO enrichment of Dlx2, E2f1 and Tsc22d4 regulons, asterisks indicate neural development-related functions. (**D**) Spatial mapping of RAS for Dlx2, E2f1 and Tsc22d4 in spots predominantly containing GABAergic neuron_2, glutamatergic neurons and neuroblasts, separately. (**E**) Proportions of spots with RAS > 0, with statistical significance determined by Pearson’s chi-squared test. **p < 0.01; ***p < 0.001; ns, not significant. (**F**) Brain region localization of differently activated regulons in two sexes

To systematically characterize gene regulatory networks affected by prenatal HE exposure, we performed SCENIC analysis [30]. Our analysis identified 18 regulons, which we compared based on their RAS between HE and VC brains (Table S8). Focusing on the top three regulons with highest RSS in each brain region (Table S9), we found seven regions exhibited sex-specific RAS differences (indicated by arrows in Fig. 6A), which were selected for further analysis; the remaining three regions showed no sex-specific differences (Figure S8).

Among these, the Dlx2, E2f1, and Tsc22d4 demonstrated the highest RAS (Fig. 6B), indicating their heightened regulatory influence. We therefore selected these three top regulons for further investigation into their potential roles. GO enrichment linked these regulons and their target genes to brain development, neuron differentiation, and neuronal stem cell maintenance (Fig. 6C). To identify the cellular basis of these regulon activity changes, we analyzed spots dominated by each region’s principal cell type and generated RAS heatmaps (Fig. 6D). The proportion of spots with positive RAS (RAS > 0) mirrored the patterns in Fig. 6B (Fig. 6E), with Dlx2 and Tsc22d4 showing male-specific differences. This cell type-specific RAS analysis indicated GABAergic neurons and neuroblasts as the main contributors to sex-dependent regulon activity changes. Spatial distributions of these differentially activated regulons are shown in Fig. 6F.

### Prenatal HE disrupts male cortical neurogenesis

**Fig. 7:**
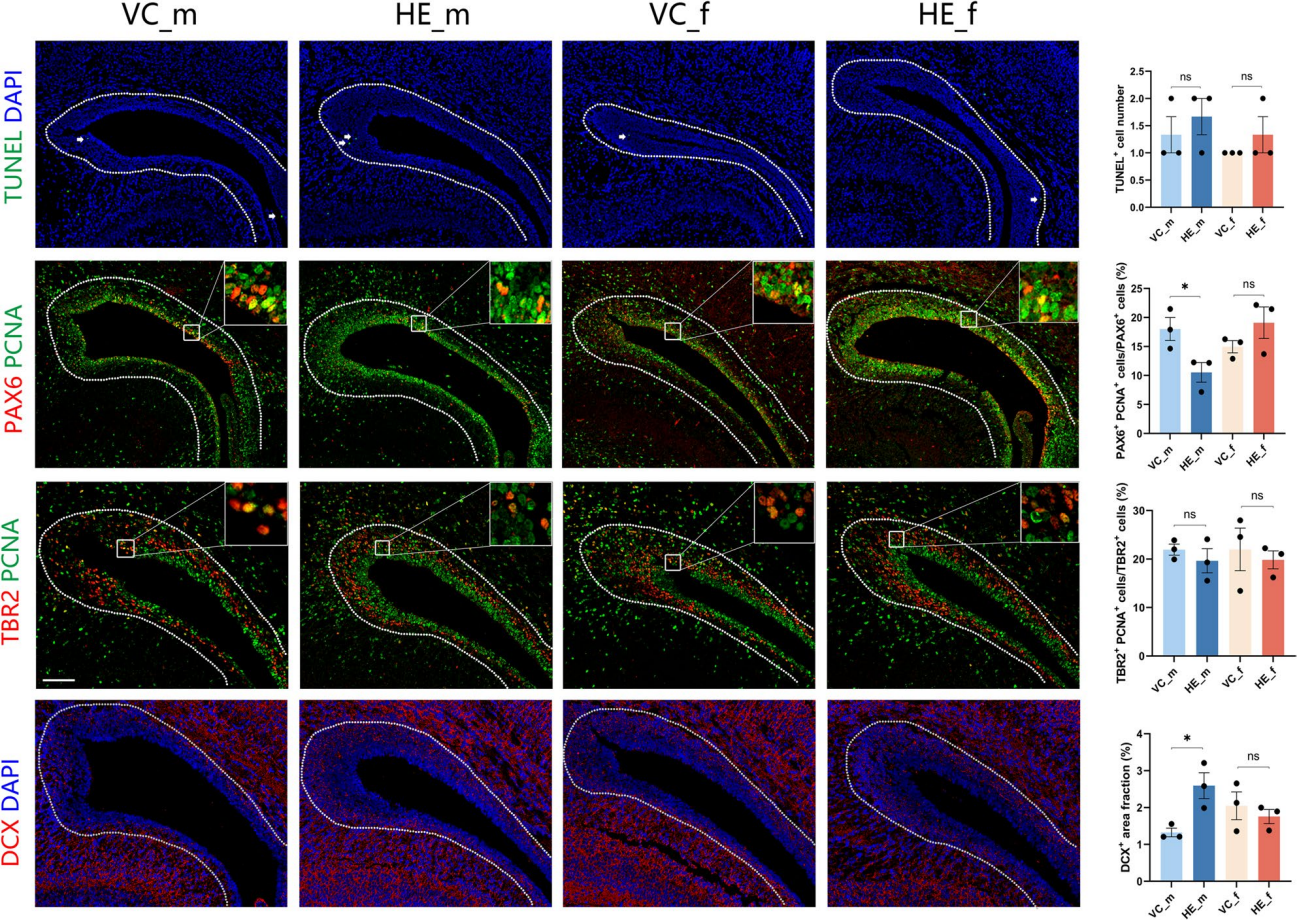
Neurogenesis assessment in DPallv. Left: Representative images of TUNEL analysis, neural progenitor (PAX6^+^PCNA^+^, TBR2^+^PCNA^+^) and immature neuron (DCX^+^) immunofluorescence in DPallv (Scale bar: 100 μm). Right: Bar graphs quantify positive cell number, cell ratio or area fraction. Data are presented as mean ± SEM, significance was determined by Student’s t-test. *p < 0.05; ns, not significant

Given that the influence on sex-biased genes, alterations in signaling pathways, and dysregulation of regulon activities converged in Dpallv - a neurogenic niche for cortical neurons - we specifically examined neurogenesis in this region (Fig. 7). No statistically significant intergroup differences were revealed in apoptotic cell (TUNEL^+^) counts. Radial glial cell (RGC) and intermediate neural progenitor (INP) in DPallv serve as primary progenitors for cortical neurons [33]. Male HE specimens exhibited a marked reduction in the ratio of proliferating RGCs (PAX6^+^PCNA^+^/PCNA^+^ cells) and an increase in immature neurons (DCX^+^), while the ratio of proliferating INPs (TBR2^+^PCNA^+^/PCNA^+^ cells) remained unaffected relative to controls. These alterations were absent in female specimens, indicating male-specific disruption of neural progenitor proliferation-differentiation balance following maternal HE exposure.

## Discussion

The intrauterine environment serves as a critical developmental window where maternal factors can induce sex-specific programming with lasting health consequences. While elevated maternal estradiol levels have been implicated in male-biased neurodevelopmental vulnerabilities [11, 13, 14], the underlying molecular mechanisms remain poorly characterized. This study provides novel insights into sex-specific neural programming by systematically mapping the transcriptional and cellular responses to prenatal high estradiol exposure in fetal brains.

The estradiol valerate treatment was administered from E5.5 to E11.5. Since previous study has reported elevated maternal estradiol during early pregnancy in fresh embryo transfer compared with spontaneous conception [9], this window was selected to specifically target early pregnancy, rather than the entire gestation. On the other hand, this is the core period of neural crest cell migration, proliferation, and differentiation [20], terminating the treatment at E11.5 allows us to attribute any observed phenotypes at E18.5 specifically to disruption during this sensitive window, rather than later processes of growth or maturation. The chosen dose of estradiol valerate (100 µg/kg/day) is clinically relevant, as it replicates the peak estradiol increase (approximately fourfold) observed in women during early pregnancy following fresh embryo transfer compared to natural conception [9]. This ensures that our model accurately reflects a specific and realistic human exposure scenario.

The selection of E18.5 as the endpoint for this analysis was aimed at optimally capturing the sustained transcriptional impact of HE on fetal brain development. At this late gestational stage, immediately prior to birth, the fundamental architecture of the mouse brain is largely complete, with major structures fully formed and key developmental processes predominantly concluded [20, 34]. Consequently, the transcriptional landscape at E18.5 is more stabilized compared to earlier stages (e.g., E15.5-E16.5), which are characterized by intense cellular dynamics and profound endogenous transcriptional fluctuations that can obscure specific responses to external insults. Later stages such as E19.5 or postnatal day 0 (P0) introduce significant confounders, including the stress of labor and the abrupt transition to extra-uterine life, which dramatically alter the transcriptome and would complicate the attribution of observed changes solely to in utero HE exposure. We focused on thalamic coronal sections, as these contain the highest density of distinct brain structures and represent a dynamic developmental stage involving multiple cell types and region-specific maturation processes [20]. Spatial clustering of our sections closely mirrored anatomical regions, including key structures such as the cortex, hippocampus, thalamus, and hypothalamus [35]. This strategy allowed comprehensive assessment of region-specific responses to HE exposure.

It is worth noting that different tissue embedding and sectioning protocols were employed in our study based on the specific requirements of each downstream assay. For spatial transcriptomics, fresh-frozen OCT-embedded tissues were sectioned at 10 μm to optimally preserve RNA integrity, as mandated by the technology. For histological analyses (TUNEL/immunofluorescence staining), tissues were paraffin-embedded and sectioned at 4 μm to achieve superior morphological resolution for precise cellular identification and quantification. Although the methods are different, this ensures that each technical route can obtain the most reliable data. The results of the two technologies are mutually confirmed and complementary, and jointly reveal our findings.

Prenatal HE exposure was observed to induce notable alterations in gene expression, with distinct DEG profiles in each brain region and between sexes. The categorization of DEGs into sex-specific and sex-concordant groups underscores that neural responses to HE are not uniform but are finely tuned by both brain region and sex. A particularly striking finding was the predominant enrichment of neural development processes—specifically within male-specific DEGs—in the hypothalamus, DPallv, and p2R regions (Fig. 2). This regional and sexual specificity nominates these areas as potential “hotspots” for the sex-biased vulnerability to early-life environmental insults, shifting the focus from a whole-brain perspective to targeted neural circuits. Notably, male-specific DEGs in DPallv were enriched in synaptic organization and neuronal differentiation functions, providing a plausible molecular underpinning for the neurogenesis alterations detected by immunostaining. This compelling convergence of transcriptomic and histological evidence strongly suggests that HE disrupts the precise developmental trajectory of this neurogenic niche for cortical neurons in males. In addition, we speculate that HE exposure might disrupt the coordinated development of the potential functional network within and between hypothalamus, DPallv, and p2R, with males being more susceptible. This disruption could ultimately manifest sex-specific behavioral phenotypes like ASD in adulthood. Future studies employing region-specific manipulations, such as CRISPR-based gene editing or chemogenetic inhibition of the identified key DEGs within these hotspots, will be crucial to formally test this causal relationship and unravel the precise mechanisms linking early transcriptomic changes to lifelong neurological consequences.

Sex-biased genes—defined by their differential expression between sexes—constitute a key molecular basis for sexual dimorphism in development. These genes display substantial variation across tissues and species, frequently exhibiting cell type-specific expression patterns [36]. Although embryonic sex-biased genes are generally expressed at lower levels than in post-pubertal stages [36], we detected significant sex-dependent expression differences in fetal brains. While prior work identified 187 male-biased and 167 female-biased genes in E15.5 whole mouse brains [37], such bulk-tissue analyses lacked regional resolution. Our study significantly extends these findings by mapping sex-biased gene expression with spatial precision across fetal brain structures. Strikingly, maternal HE exerted a broader regulatory effect on sex-biased genes in male fetal brains than in females (Fig. 3J), it suggests that the developing male brain may be intrinsically more vulnerable to prenatal HE exposure. This finding is consistent with a growing body of evidence indicating that male brains can exhibit greater susceptibility to a variety of early-life neurodevelopmental insults. For instance, in a model of *Arid1b* dysregulation, neuroanatomical alterations manifested earlier in males than in females [38]. This also aligns with established evidence that estrogen modulates male-biased gene expression via ERα in brain regions governing sex-typical behavior [39]. However, our findings uniquely demonstrate that these effects are mediated through maternal estradiol rather than direct estrogen exposure. A particularly significant observation was the predominant downregulation of male-biased DEGs in HE-exposed brain regions, this pattern suggests a disruption of the masculinization program, potentially leading to a partial “feminization” of gene expression. Immunostaining validated the expression of three representative sex-biased genes affected by HE in their corresponding brain regions. TTR has been established as neuroprotective with increased expression in the prefrontal cortex of male but not female patients with depression [40]. Its downregulation in the CSPall of HE-exposed males may thus indicate a compromised anti-stress mechanism. This hypothesis merits further investigation through behavioral stress assays and quantification of TTR protein levels in the affected region. MARCKS, which modulates cytoskeletal dynamics, phospholipid signaling, organelle homeostasis, and cross-talk with other signaling pathways, plays a key role in cerebral cortex development [41, 42]. As a substrate of protein kinase C (PKC), although direct evidence for sex-specific regulation of MARCKS in the brain remains limited, it may be indirectly influenced by sex hormones via the PKC pathway, which is itself hormonally sensitive [43]. During cerebral cortex development, TNC regulates neuronal radial migration and morphological maturation by forming a physical scaffold, while also serving as an extracellular matrix glycoprotein to guide neuronal migration and maintain the microenvironment of ventricular stem cells [44, 45]. The observed downregulation of TNC in HE-exposed female DPallv suggests possible impairments in neuronal migration or maturation, though further functional validation is needed. Collectively, these findings not only nominate sex-biased genes as mediators of sex-specific vulnerability but also establish a new landscape for understanding how maternal endocrine status actively shapes fetal brain sexual dimorphism.

Building upon established spatial profiles of fetal mouse brain cell types [20], we enhanced resolution by integrating public paired-end scRNA-seq data of E18 mouse brains. Our analysis successfully mapped nine major cell types, with the exception of microglia which are predominantly localized to midbrain and hindbrain regions [20]. We identified two distinct GABAergic neuron populations based on their marker gene expression. While multiple GABAergic neuron subtypes (e.g., basket cells, chandelier cells, Martinotti cells) have been characterized in adult mouse brains [46], their fetal counterparts remain undefined. Our findings suggest that GABAergic neuron_1 and GABAergic neuron_2 may represent distinct fetal subtypes, though this requires experimental validation. The category “other neurons” included all non-GABAergic, non-glutamatergic neuronal populations, which were not further subclassified due to the absence of definitive marker genes. Glutamatergic neurons, GABAergic neurons, neuroblasts, and other neurons emerged as the most abundant cell types. Their spatial distribution strongly correlated with anatomical brain regions, reinforcing the region-specific nature of cellular clustering patterns.

The identification of HE-affected sex-biased DEGs in specific neural cell types offers critical insights into the cellular mechanisms underlying sex-dimorphic responses to HE exposure. Among these, *Avp*, *Mdga1*, and *Ttr* have been discussed in their corresponding neural cells in existing literature. *Avp*, a neuropeptide modulating homeostatic functions and social behaviors, demonstrates male-biased expression [47], and interacts with GABAergic signaling in blood pressure regulation [48] and thermoregulation [49]. *Mdga1*, involved in neural development and inhibitory synapse formation [50, 51], emerges as a potentially novel sex-biased gene, as its sexually dimorphic expression has not been previously reported. Thyroid hormone binding and distribution protein *Ttr* [52], crucial for neural stem cell fate determination [53], showed male-biased upregulation in neuroblasts, suggesting a potential mechanism for HE-induced neural developmental alterations. These findings highlight the need for further investigation to fully elucidate the roles of sex-biased genes in specific cell types and their modulation by HE exposure.

During brain development, signaling pathways orchestrate the transmission of information between neural cells and profoundly influence the formation and functional maintenance of the nervous system. Since the physiological functions within a single brain region are relatively uniform, we concentrated on signaling pathways within each brain region. It was notable that a considerable number of signaling pathways demonstrated a sexually opposite change in interaction number upon HE exposure. Literature review confirms that these identified pathways are critically involved in brain development: FLRT proteins regulate neuronal migration and FGF signaling [54–56]; CDH pathway contributes to neural layer organization and circuit formation [57]; SEMA4/5 pathways govern axon guidance, neuronal migration, and synaptogenesis [58, 59]; APP signaling modulates glial differentiation and neurogenesis [60]; glycine mediates excitatory neurotransmission and neurogenic processes [61, 62]; LAMININ supports neural cell migration and pathway development [63]; CSPG4 facilitates oligodendrocyte precursor morphogenesis [64]; NECTIN and netrin proteins are essential for axon guidance [65–67]; EPHA signaling controls neural morphogenesis and apoptosis [68–70], and PTN promotes dendritic development [71]. While these findings highlight the complexity of sex-dimorphic intercellular signaling, the underlying mechanisms warrant further investigation, representing an important direction for future research.

Transcription factors shape the sex differences in brain development through multiple mechanisms such as hormone response [72], neural circuit specificity regulation [39] and epigenetic integration [73], making the identification of key regulons essential for understanding sex dimorphism in response to HE exposure. We used SCENIC to identify specific regulons by first detecting co-expression modules around transcription factors and then refining them through cis-regulatory motif enrichment analysis. The activity of each resulting regulon was quantified in individual cells using the AUCell algorithm. A high RAS signifies that the entire regulatory network is highly active within a cell, pinpointing key regulons that define specific cell states and warranting further investigation. Our analysis uncovered sex-specific changes in the activity of seven regulons following HE exposure, among them three key regulons with prominent higher RAS-Dlx2, E2f1, and Tsc22d4-presented distinct cellular associations in GABAergic neurons, glutamatergic neurons, and neuroblasts, respectively. Dlx2, a homeodomain transcription factor critical for the development of GABAergic neurons [74], showed attenuated activity in the HE_m prethalamus, suggesting potential impairment in GABAergic interneuron differentiation or maintenance. The paradoxical observation of increased E2f1 regulon activity and reduced RGC proliferation in the HE_m Dpallv presents an intriguing case of the dual functionality of this transcription factor. While E2f1 is classically characterized as a cell cycle promoter [75], its aberrant activation could also drive cell-cycle exit and differentiation [76], potentially explaining the decreased proliferating RGCs observed in the HE_m Dpallv. It is noteworthy that while the RAS of E2f1 was markedly elevated in HE_m relative to VC_m brain (Fig. 6B), the proportion of E2f1-positive spots among those identified as glutamatergic neurons did not exhibit a statistically significant difference between the two groups (Fig. 6E), indicating additional cellular targets beyond glutamatergic neurons. The discovery of enhanced Tsc22d4 regulon activity in male p2R neuroblasts represents a novel finding, as this transcription factor’s role in fetal neural development remains largely unexplored. While Tsc22d4 has been implicated in cerebellar granule neuron differentiation during postnatal stages [77], our results suggest potential involvement in thalamic neural network formation during embryonic development. Given neuroblasts’ fundamental contribution to thalamic circuitry establishment [78, 79], this finding provides new clues for understanding how maternal hormonal milieu may shape subcortical development. The functional enrichment of these regulons highlighted the diversity and complexity of transcription factors in neural development and their roles in shaping the cell types and functions of different brain regions. Collectively, the dysregulation of Dlx2, E2f1, and Tsc22d4 regulons in response to HE exposure paints a coherent picture of disrupted neurodevelopmental trajectories in males. We propose a convergent model wherein these perturbations collectively impair the balance between proliferation, differentiation, and circuit formation in a sex-specific manner. The attenuation of Dlx2, a master regulator of inhibitory neuron development, alongside the paradoxical activation of E2f1, which may force premature cell-cycle exit in progenitors, suggests a dual hit on the generation and specification of neuronal populations. This is further compounded by the aberrant activation of Tsc22d4 in neuroblasts, potentially altering the maturation of thalamic circuits. Thus, HE exposure does not merely cause random transcriptional noise but appears to co-opt key nodes of the neurodevelopmental program, leading to a coordinated failure in the establishment of specific neural networks in the male brain, which may underlie the observed sex-biased behavioral outcomes. Future studies employing in utero electroporation to knock down or overexpress Dlx2, E2f1, or Tsc22d4 in their specific brain regions could directly test their necessity and sufficiency in mediating HE’s effects on RGC proliferation and neuron maturation.

**Fig. 8:**
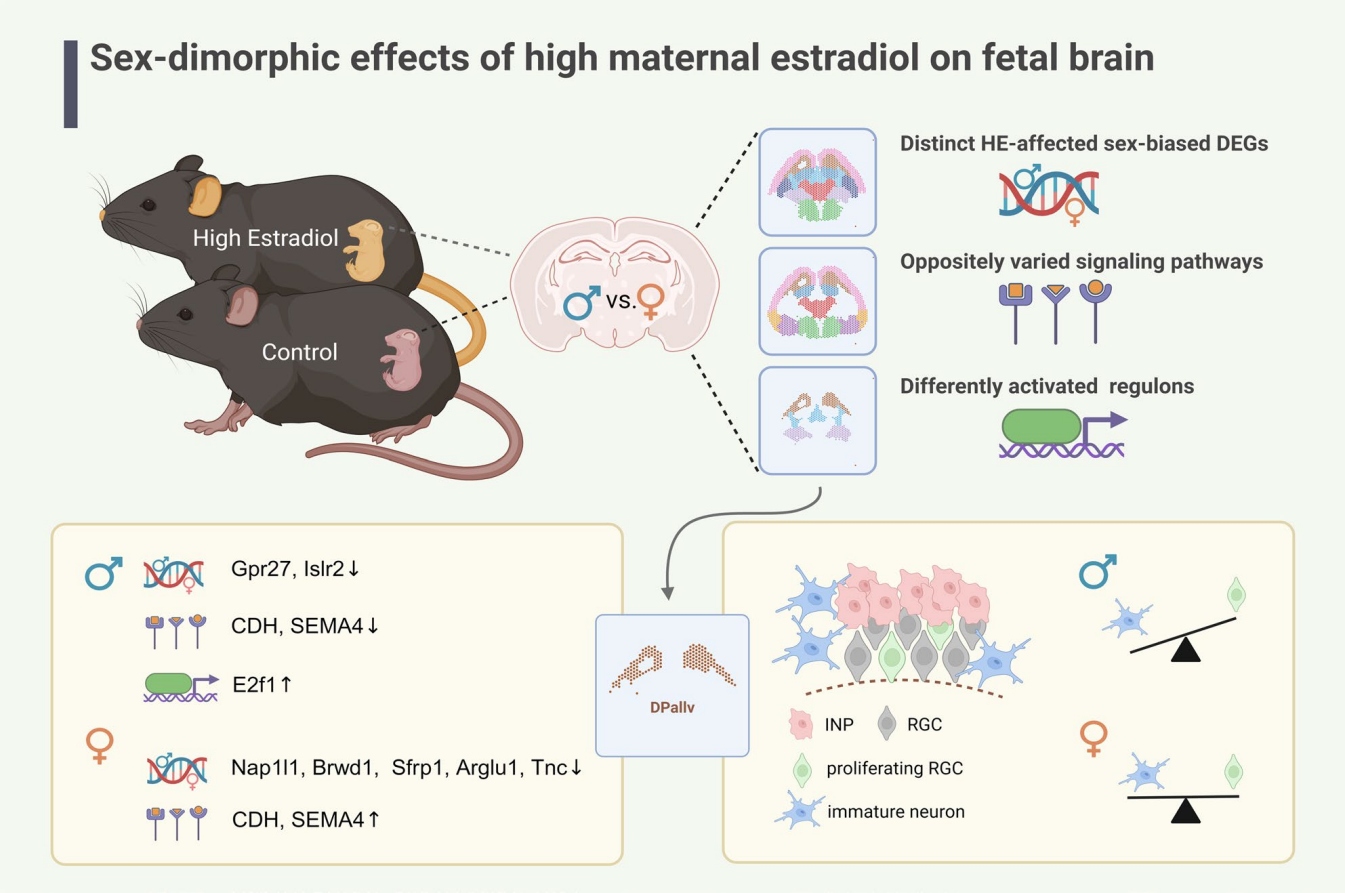
Mechanistic overview of sex-dimorphic responses to prenatal HE exposure in fetal brains

Throughout the above studies, the sex-dimorphic effect of high maternal estradiol on the fetal mouse brain was reflected in distinct HE-affected sex-biased genes, oppositely changed signaling pathways and differentially regulated regulon activities. These aspects involved different brain regions separately, with DPallv emerging as a common site of interest, probably a particularly vulnerable site (Fig. 8). This region presented different HE-affected male biased (Gpr27, Islr2) and female biased (Nap1l1, Brwd1, Sfrp1, Arglu1, Tnc) DEGs, sexually-opposite change of intercellular interactions in CDH and SEMA4 pathways, and male-specific increase of E2f1 regulon activity. The male-biased neurogenesis alteration in DPallv might be a synergistic result of the molecular perturbations listed above, although more definitive evidence is required to elucidate the specific mechanisms. We observed a reduced proportion of proliferating RGCs and an increased number of immature neurons in HE_m DPallv, reflecting a “premature” cell cycle exit and accelerated differentiation. It has been proved that the balance between proliferation and differentiation of neural stem cells is crucial for maintaining the neural progenitor cell pool, ensuring the production of appropriate quantities and types of neurogenic cells during brain development, its disruption may lead to brain malformations or functional impairments [80–82]. While direct clinical evidence linking maternal estradiol to sex-specific interference of cortical neurogenesis remains limited, our findings align with reports of altered cortical folding in ART-conceived fetuses following fresh embryo transfer, potentially attributable to altered intrauterine hormonal environments as represented by maternal estradiol [83]. This study provides mechanistic insights into male-specific cortical reorganization under high maternal estradiol conditions and highlights the need for further investigation of its long-term neurodevelopmental impacts.

While our study and much of the extant literature have focused on iatrogenic HE resulting from ovarian stimulation in ART, it is important to consider other potential etiologies. Notably, exposure to environmental endocrine-disrupting chemicals (EDCs), such as bisphenol A (BPA) and phthalates, represents a significant non-iatrogenic source. These compounds, which structurally mimic estradiol, can act as xenoestrogens by competitively binding to estrogen receptors and activating downstream signaling pathways, potentially contributing to elevated maternal estrogen levels [84, 85]. Furthermore, individual genetic susceptibility may play a role. Polymorphisms in genes involved in estrogen biosynthesis (e.g., *CYP19A1*) [86], metabolism, or receptor function (e.g., ESR1/2) [87–89] could predispose some women to heightened estradiol responses under various physiological or environmental conditions. Future studies that integrate assessments of EDC exposure and genetic markers are warranted to provide a more comprehensive understanding of the potential sex-dimorphic effect of maternal HE on fetal development.

Although this study was conducted using a mouse model, its findings hold profound implications for human health. As HE-exposure in utero increases risk of ASD in male offspring [13, 14], we cross-referenced our list of HE-induced male-biased DEGs with the Simons Foundation Autism Research Initiative (SFARI) Gene database (accessed: October 2025) [90]. While a direct overlap with the highest-confidence SFARI genes was limited, we observed dysregulation of genes with strong functional links to ASD mechanisms. As the database recorded, disruptions in the AVP system (e.g., V1aR knockout) are directly linked to impaired social behavior in models [91]. The alteration of *Avp* in our study provides a direct molecular bridge between prenatal HE exposure and a core ASD-related behavioral domain. Although our study identified *Mdga1* while literature more strongly links *Mdga2* to autism [92, 93], both genes belong to the same MDGA family, which are known critical regulators of synaptic development and cortical circuit formation. Furthermore, these transcriptional disruptions occurred in brain regions fundamental to social and repetitive behaviors, such as the cortex and hypothalamus. This convergence of gene function and neuroanatomy provides a potential pathway through which prenatal HE may contribute to a male-biased risk for neurodevelopmental disorders. In addition, the key neurodevelopmental pathways LAMININ, Netrin, SEMA, EPHA, and Glycine we identified demonstrates high conservation between mouse and human [94–99], suggesting our findings may provide experimental explanations for the etiology of sex-specific human neurodevelopment disorders. By pinpointing specific dysregulated pathways, our model provides a platform for future testing of neuroprotective interventions during vulnerable windows. We acknowledge that translation requires validation in human models and correlation with clinical cohorts. However, these findings establish important mechanistic foundations for understanding maternal hormone’s impacts on fetal neurodevelopment and outline concrete steps toward potential clinical applications.

Building on our findings, future research should prioritize longitudinal analysis of sex-specific vulnerabilities. A crucial next step involves integrating single-cell RNA sequencing with spatial transcriptomics to track the dynamic, cell-type-specific molecular signatures in key brain regions across developmental stages - from embryonic periods through postnatal maturation. This approach will reveal how sex differences in neuronal and glial populations emerge and evolve in response to HE exposure, identifying critical windows of vulnerability. Mechanistic studies should then employ genetic tools to perturb identified sex-specific pathways in relevant cell types and developmental windows. Longitudinal behavioral assessments must be designed to detect how these divergent developmental trajectories manifest as sex-biased cognitive or social deficits in adulthood. Finally, generating developmental brain organoids from male and female iPSCs will enable testing of conserved sex-specific mechanisms in a human model system. This comprehensive, sex-specific framework - spanning molecular dynamics, functional validation, and cross-species translation - will be essential for developing precisely targeted neuroprotective strategies.

## Conclusions

This study establishes the first spatial transcriptomic atlas characterizing sex-dimorphic responses to prenatal high-estradiol exposure in the developing mouse brain, revealing novel molecular mechanisms underlying developmental programming. Our identification of regionally selective vulnerabilities and sexually dimorphic regulatory networks underscores the critical need for sex-stratified therapeutic approaches in neurodevelopmental disorders. These findings carry important clinical implications, informing both the optimization of assisted reproductive technologies and the development of targeted interventions to ameliorate the neurodevelopmental consequences of gestational endocrine disruption.

## Supplementary Information

Supplementary Material 1: Figure S1. Anatomical annotation of spatial transcriptomics clusters.

Supplementary Material 2: Figure S2. Functional enrichment of HE-induced DEGs in selected brain regions (Part 1).

Supplementary Material 3: Figure S3. Functional enrichment of HE-induced DEGs in selected brain regions (Part 2).

Supplementary Material 4: Figure S4. Cell type annotation using specific markers.

Supplementary Material 5: Figure S5. Comprehensive visualization of information flow across 52 signaling pathways in brain slices.

Supplementary Material 6: Figure S6. Intra-regional signaling with sex-opposed changes.

Supplementary Material 7: Figure S7. Regional pathway gene score distribution.

Supplementary Material 8: Figure S8. Regulon activity in selected brain regions.

Supplementary Material 9

Supplementary Material 10: Table S1. Findallmarker for spatial transcriptome.

Supplementary Material 11: Table S2. HE induced DEGs.

Supplementary Material 12: Table S3. GO enrichment of HE-induced DEGs.

Supplementary Material 13: Table S4. Findallmarker for single-cell RNA-seq.

Supplementary Material 14: Table S5. Markers for cell type identification.

Supplementary Material 15: Table S6. RCTD.

Supplementary Material 16: Table S7. Ligand-receptor interaction networks.

Supplementary Material 17: Table S8. RAS.

Supplementary Material 18: Table S9. RSS.

## Data Availability

The spatial transcriptome data are available at National Center for Biotechnology Information repository with the accession number PRJNA1172454 (The reviewer link is [https://dataview.ncbi.nlm.nih.gov/object/PRJNA1172454?reviewer=pria6p4e8slk9ohlu6gp290vh4]). The datasets generated during this study are included in supplementary information files.
