# Supplementary figures and images for: Sex-dimorphic reprogramming of fetal mouse brain development by maternal estradiol excess
Source: Biol Sex Differ. 2025 Dec 2;17:1. doi: 10.1186/s13293-025-00792-7 (PMC12777311; doi:10.1186/s13293-025-00792-7)

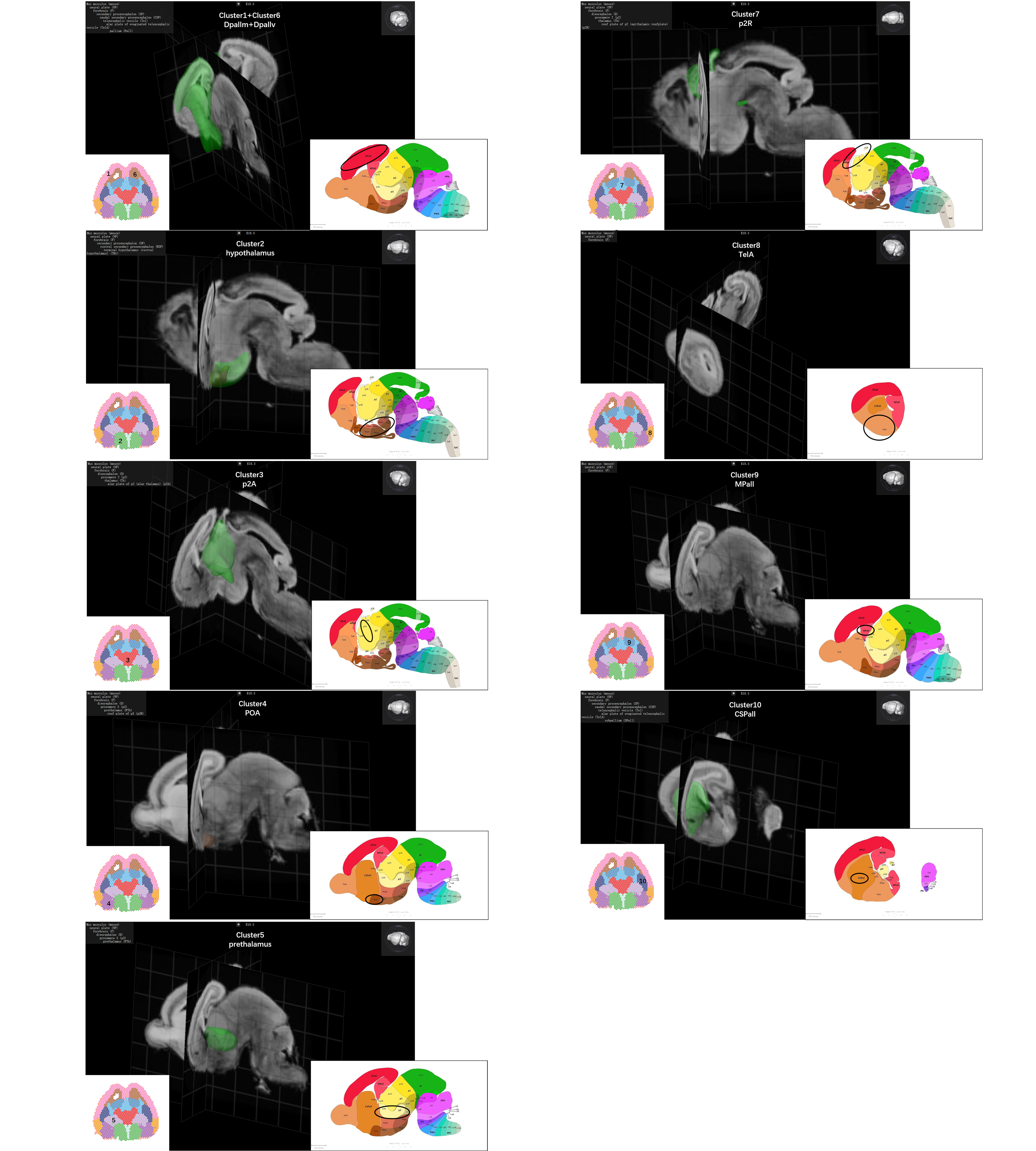

Supplement: Supplementary file 1 — Supplementary Material 1: Figure S1. Anatomical annotation of spatial transcriptomics clusters. [file 13293_2025_792_MOESM1_ESM.tif]

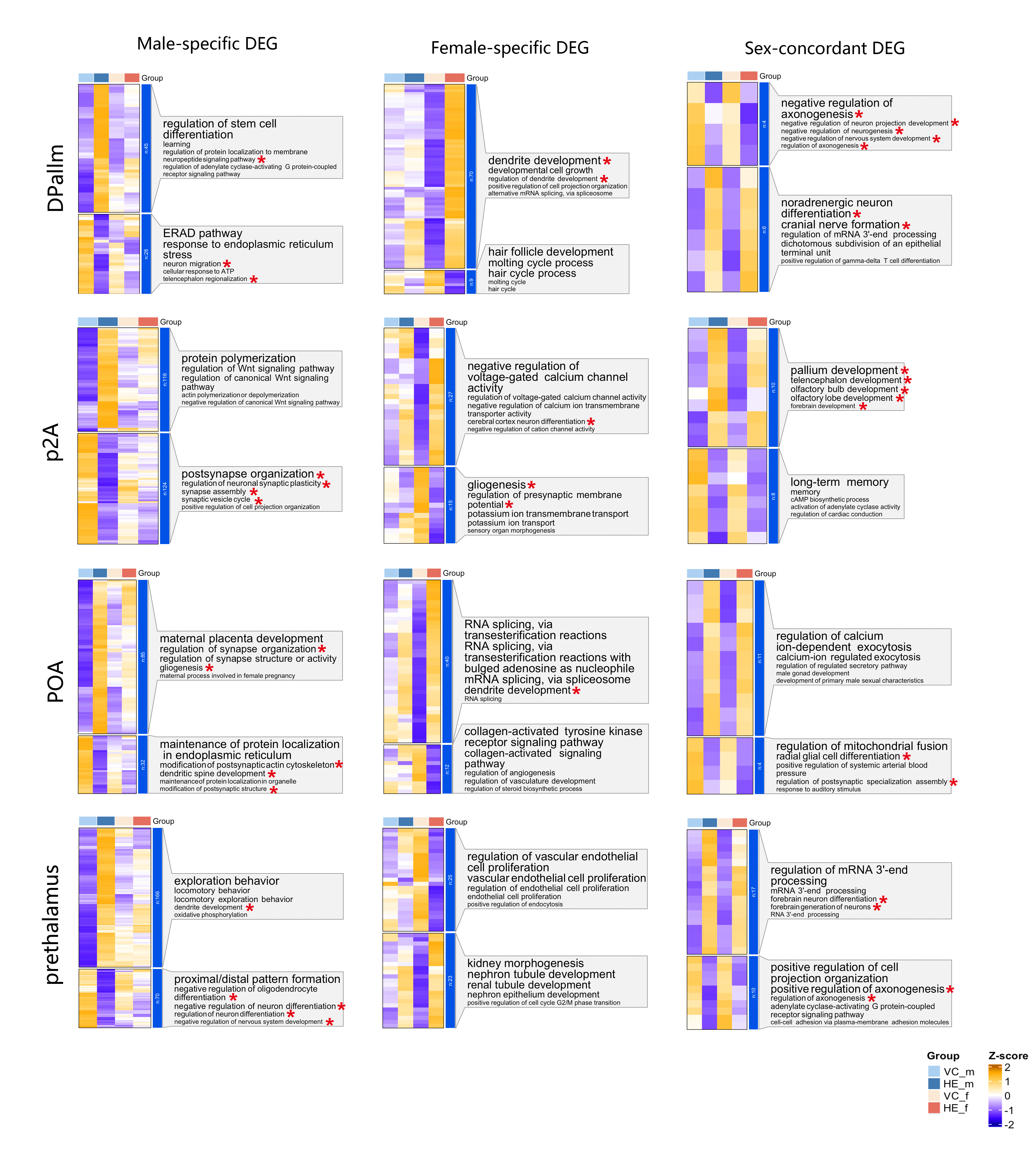

Supplement: Supplementary file 2 — Supplementary Material 2: Figure S2. Functional enrichment of HE-induced DEGs in selected brain regions (Part 1). [file 13293_2025_792_MOESM2_ESM.tif]

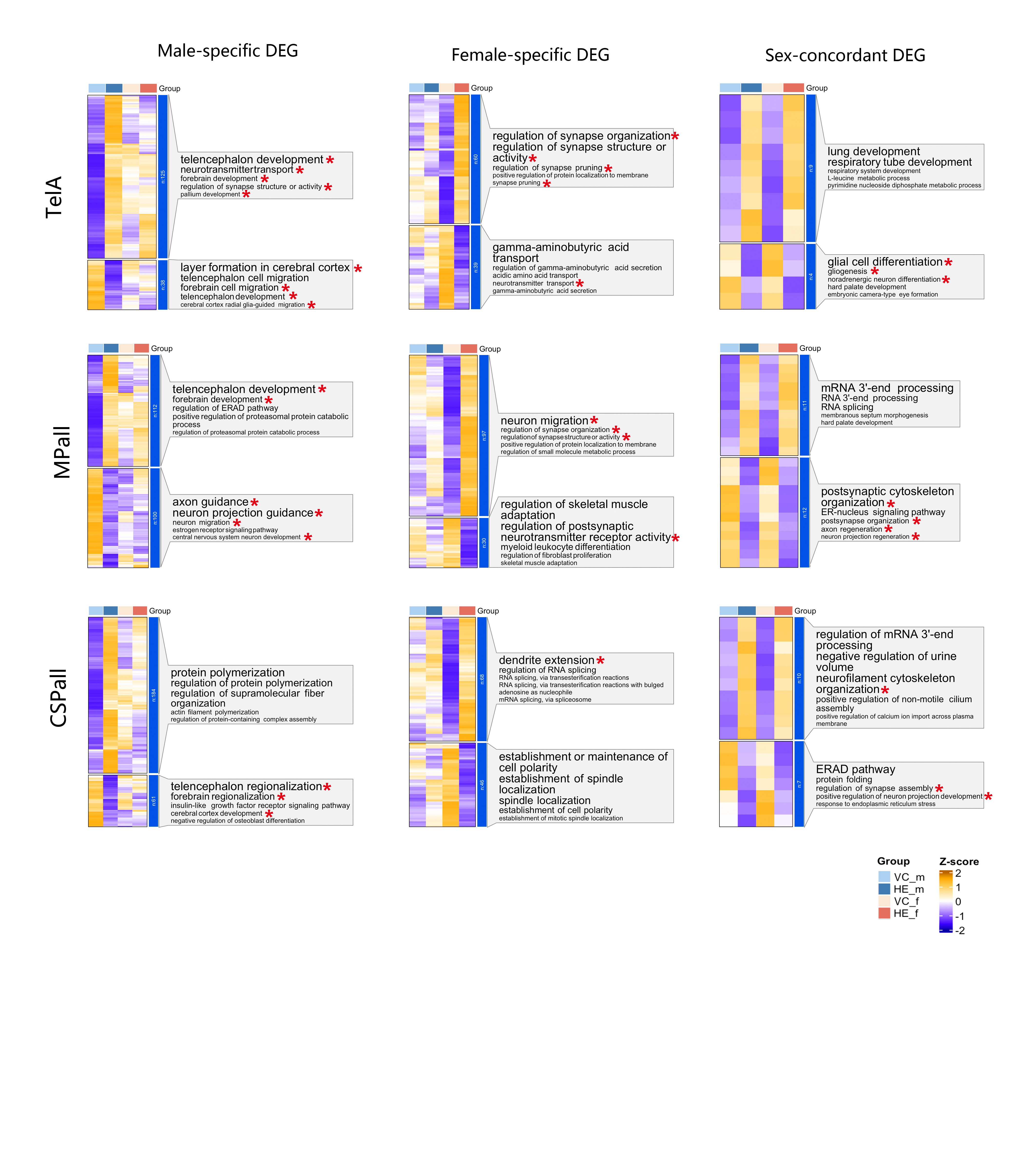

Supplement: Supplementary file 3 — Supplementary Material 3: Figure S3. Functional enrichment of HE-induced DEGs in selected brain regions (Part 2). [file 13293_2025_792_MOESM3_ESM.tif]

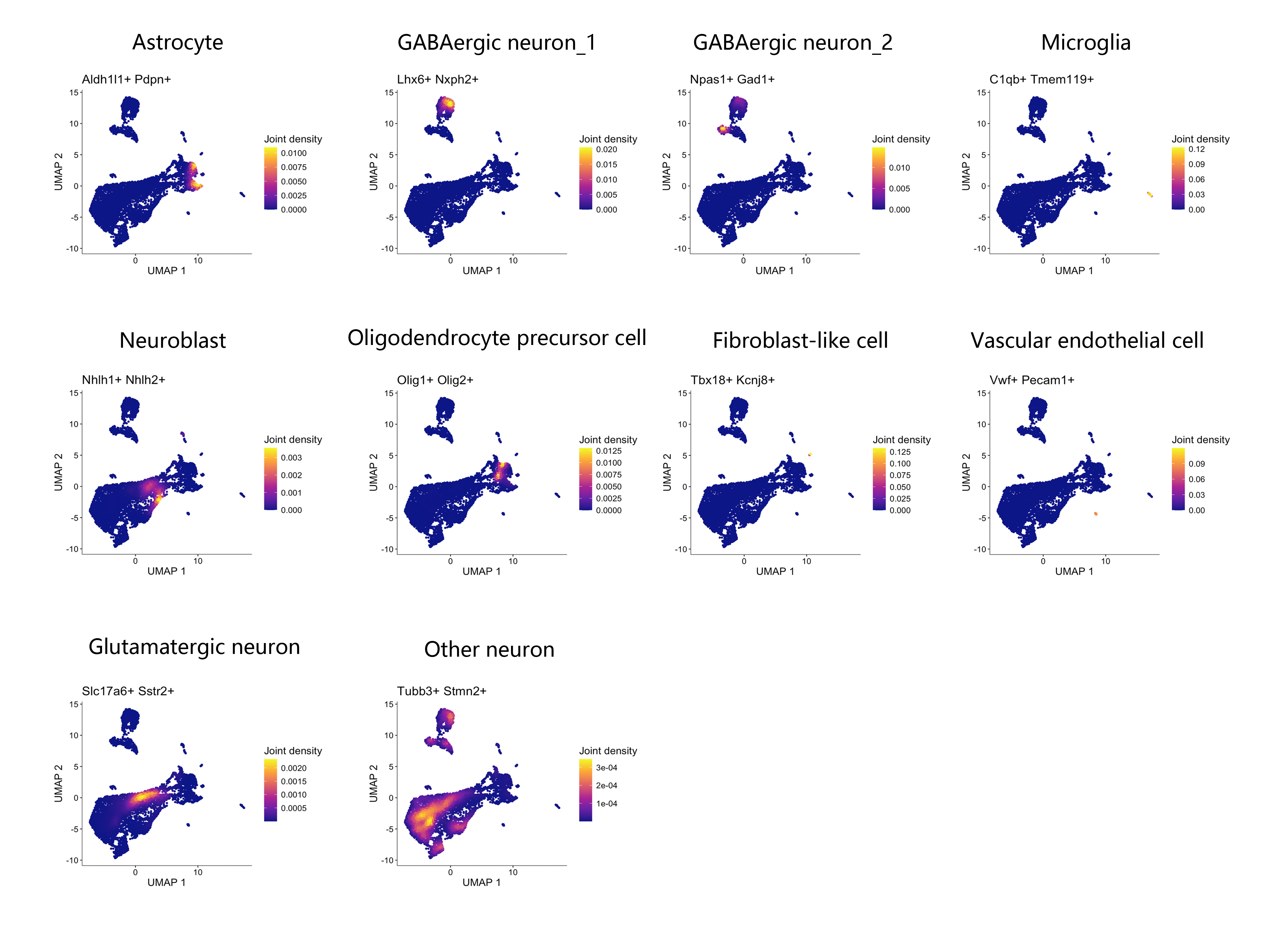

Supplement: Supplementary file 4 — Supplementary Material 4: Figure S4. Cell type annotation using specific markers. [file 13293_2025_792_MOESM4_ESM.tif]

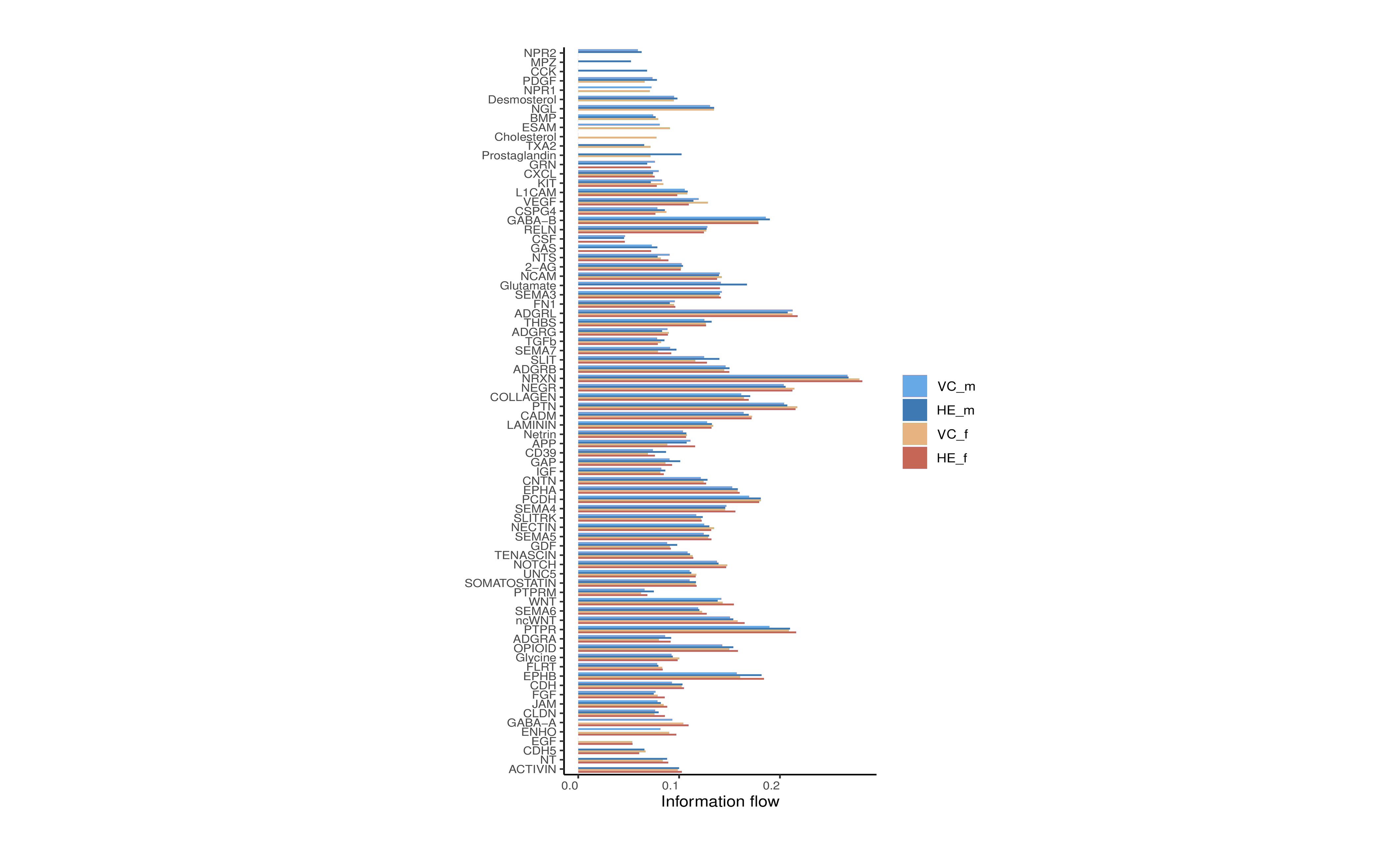

Supplement: Supplementary file 5 — Supplementary Material 5: Figure S5. Comprehensive visualization of information flow across 52 signaling pathways in brain slices. [file 13293_2025_792_MOESM5_ESM.tif]

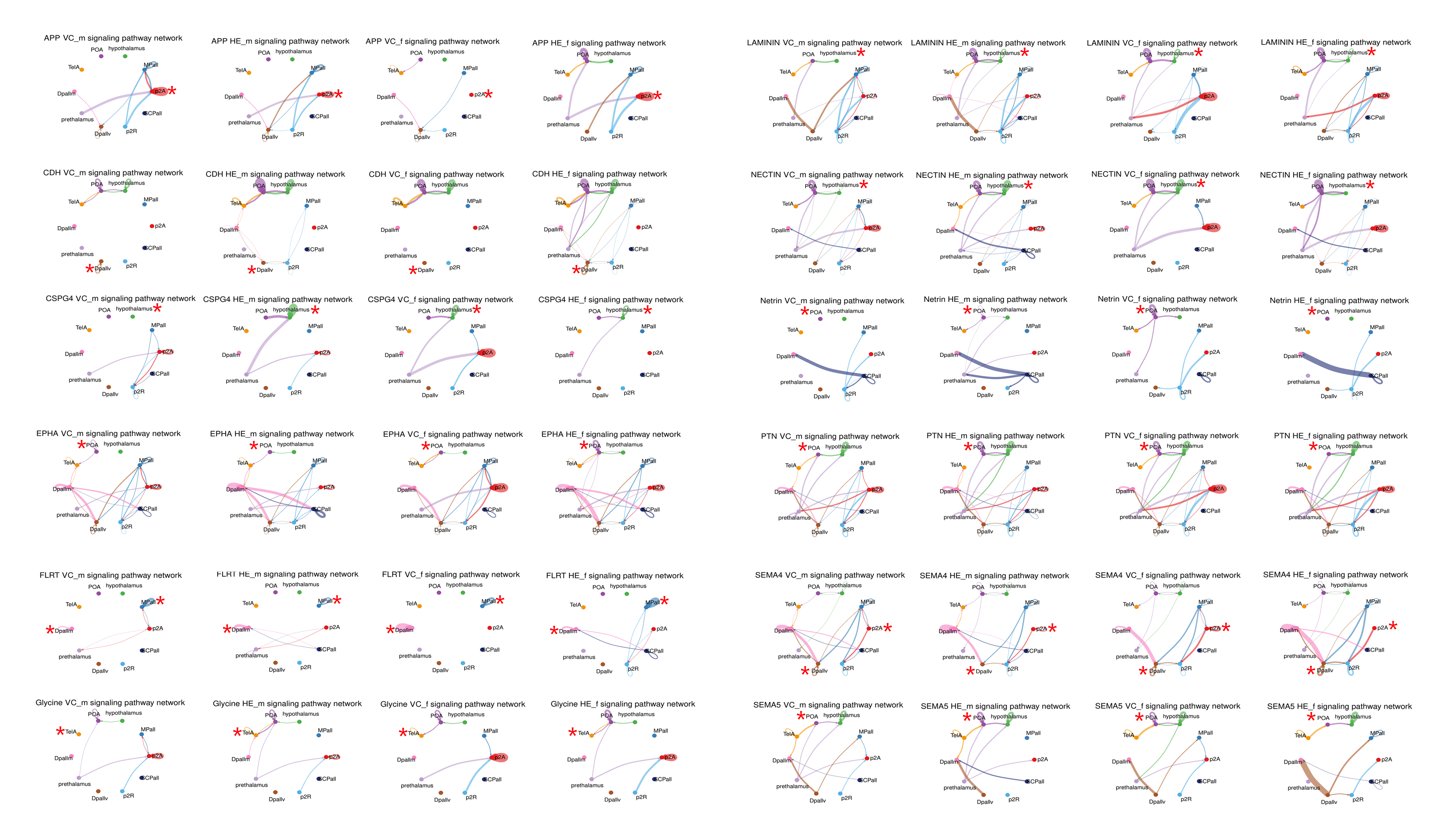

Supplement: Supplementary file 6 — Supplementary Material 6: Figure S6. Intra-regional signaling with sex-opposed changes. [file 13293_2025_792_MOESM6_ESM.tif]

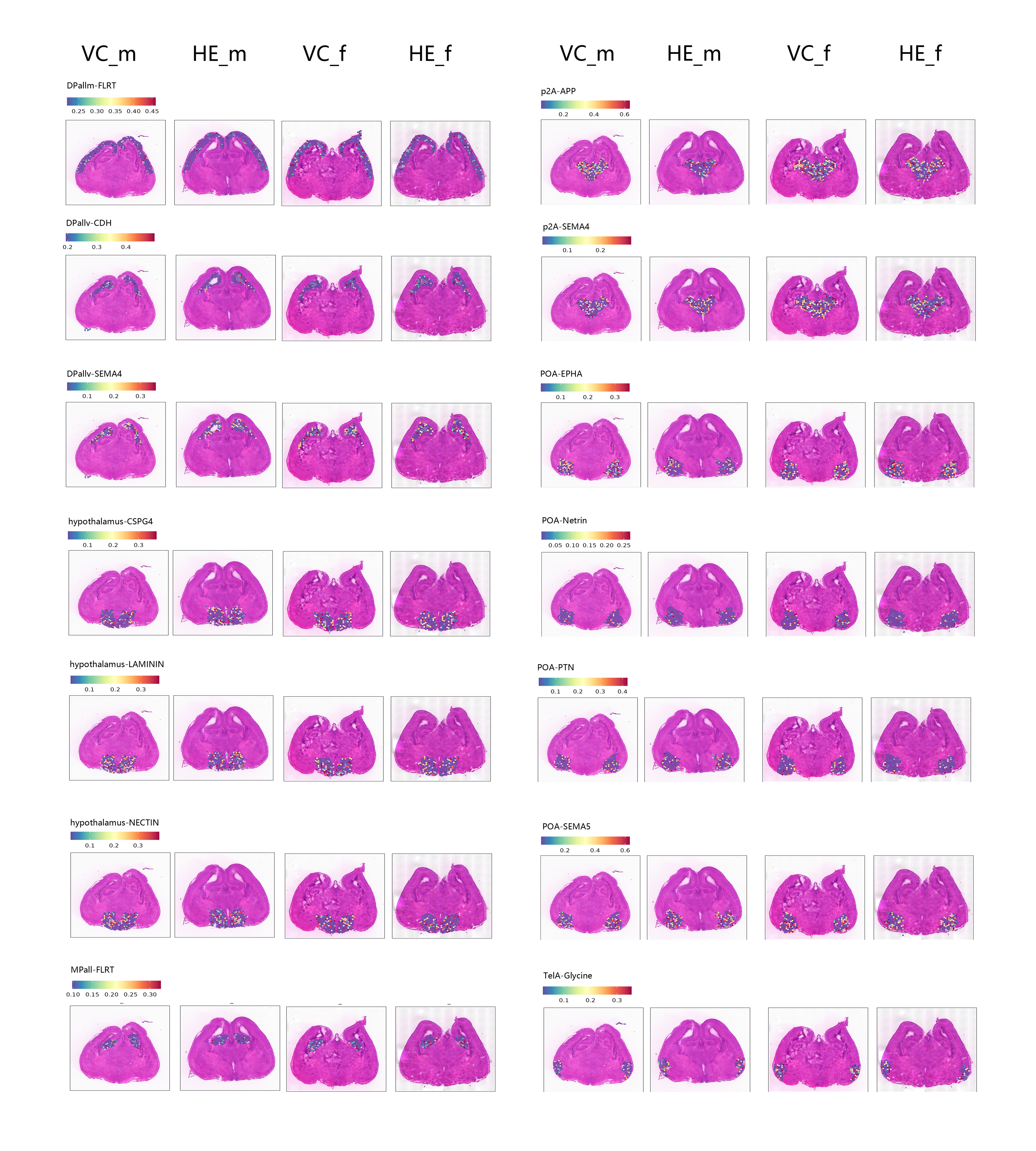

Supplement: Supplementary file 7 — Supplementary Material 7: Figure S7. Regional pathway gene score distribution. [file 13293_2025_792_MOESM7_ESM.tif]

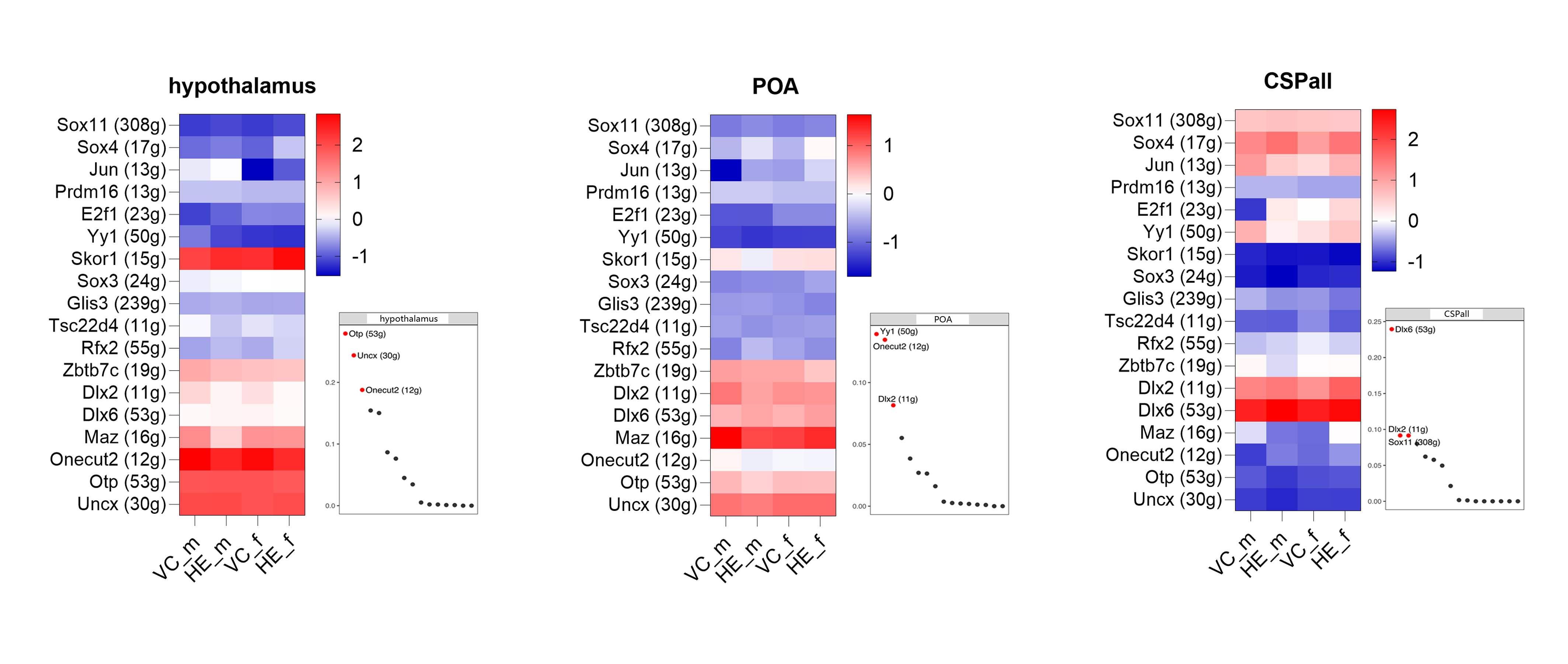

Supplement: Supplementary file 8 — Supplementary Material 8: Figure S8. Regulon activity in selected brain regions. [file 13293_2025_792_MOESM8_ESM.tif]
