# Supplementary material for: Sex-dimorphic reprogramming of fetal mouse brain development by maternal estradiol excess
Source: Biol Sex Differ. 2025 Dec 2;17:1. doi: 10.1186/s13293-025-00792-7 (PMC12777311; doi:10.1186/s13293-025-00792-7)
Supplement: Supplementary file 9 — Supplementary Material 9 [file 13293_2025_792_MOESM9_ESM.docx]

**Supplementary figure legend**

**Supplementary Figure 1. Anatomical annotation of spatial transcriptomics clusters.** Spatial clusters were mapped to corresponding fetal brain regions using the E18.5 Allen Brain Atlas and Brain Explorer 2 as reference.

**Supplementary Figure 2. Functional enrichment of HE-induced DEGs in selected brain regions (Part 1)**. GO enrichment analysis of sex-specific and sex-concordant DEGs in DPallm, p2A, POA and prethalamus, asterisks indicate neural development-related functions.

**Supplementary Figure 3. Functional enrichment of HE-induced DEGs in selected brain regions (Part 2)**. GO enrichment analysis of sex-specific and sex-concordant DEGs in TelA, MPall and CSPall, asterisks indicate neural development-related functions.

**Supplementary Figure 4. Cell type annotation using specific markers.** Density plots illustrate the expression patterns of marker genes used for annotation. Color bar reflects joint density.

**Supplementary Figure 5. Comprehensive visualization of information flow across 52 signaling pathways in brain slices.**

**Supplementary Figure 6. Intraregional signaling with sex-opposed changes.** Network visualization of 12 signaling pathways displaying sex-opposed changes in intraregional ligand-receptor interaction numbers in HE vs. VC brains (red: up-regulation; blue: down-regulation; line thickness: magnitude of change), asterisks indicate brain regions presenting sex-opposed changes.

**Supplementary Figure 7. Regional pathway gene score distribution.** Heatmap depicting gene scores for the 12 sex-oppositely changed pathways in corresponding brain regions. Color bar reflects gene score.

**Supplementary Figure 8. Regulon activity in selected brain regions.** Left: Heatmap of RAS in brain regions without sex-dimorphic RAS alterations. Right: Top three brain region-specific regulons ranked by RSS.
